# Human TET2-mutant clonal hematopoiesis expansion is driven by distinct inflammatory signaling responses in stem cells versus myeloid progeny

**DOI:** 10.1158/2643-3230.BCD-25-0070

**Published:** 2026-03-04

**Authors:** Hector Huerga Encabo, Giuseppe D'Agostino, Katherine Sturgess, Alice E. Lord, Eric D. Jong, Alessandra Ferrelli, Aneesh Sharma, Syed A. Mian, Khadidja Habel, Miriam Llorian-Sopena, Amirtha Priya Ramesh, Fatihah Mohamad Nor, Helene Foissner, Manuel Garcia-Albornoz, Lynette Graver, Despoina Papazoglou, Steven Ngo, Fernando Anjos-Afonso, Linda Ariza-McNaughton, Gabriella Ficz, Dominique Bonnet

**Affiliations:** 1 Haematopoietic Stem Cell Laboratory, The Francis Crick Institute, London, NW1 1AT, UK; 2 Plasticell Limited, Stevenage Bioscience Catalyst, Stevenage, UK; 3 Centre for Haemato-Oncology, Barts Cancer Institute, John Vane Science Centre, Charterhouse Square, Queen Mary University of London, London EC1M 6BQ, United Kingdom; 4 Bioinformatics and Biostatistics, The Francis Crick Institute, 1 Midland Road, London, NW1 1AT, UK

## Abstract

Clonal hematopoiesis (CH) increases with age and is associated with severe outcome in the course of infections or tumor development. Understanding the environmental conditions that favor mutant clones and the CH-immune system response to such environments is key to designing therapeutic strategies to stall the expansion of mutant clones and the development of CH-associated pathologies. Using human cells, we unravel a cell-specific and opposite impact of *TET2* mutations on hematopoietic stem and progenitor cells (HSPC) compared to their myeloid progeny. Multi-omic analyses reveal that TET2-mutant HSPCs exhibit intrinsic epigenetic silencing of AP-1 transcription factors and a blunted transcriptional adaptation to systemic inflammation. Conversely, monocyte-macrophage trajectory derived from TET2^Mut^ HSCs contributes to exacerbated inflammation. Together, these findings reconcile how TET2-mutant CH can simultaneously promote increased stemness within the HSPC compartment and heightened inflammation through its myeloid progeny, providing mechanistic insight into how TET2-CH expands under inflammatory stress.

## Introduction

The hematopoietic system is composed of many different hematopoietic stem cell clones. Such polyclonal diversity shrinks with aging, as individual clones become dominant. Clonal hematopoiesis (CH), characterized by the expansion of mutant HSCs and their progenies, has become model of choice to study somatic evolution with aging and to investigate how it is influenced by environmental stressors (1–4). Among the genes implicated in CH, mutations in *TET2* have gained interest due to their high frequency in the elderly population and their association with diverse human pathologies (5–12). Therefore, understanding the environmental cues and immune dysregulations associated with *TET2*-mutated CH (TET2-CH) remain a burning question (13,14). On one hand, extensive work with *Tet2*-KO mouse models have identified hematological stressors, such as aging, infection, and bone marrow transplant, that favor the expansion of TET2-CH (15–18), but supporting evidence in the human cell context remains scarce (19,20). On the other hand, we and others have contributed to the characterization of the lineage differentiation bias observed in *TET2* mutant human hematopoietic stem/progenitor cells (TET2^Mut^ HSPCs), showing the development and immune functions of mature human blood cell types (19,21,22). However, a complete understanding of the changes initiated by TET2 loss-of-function mutations within the HSPC population and their propagation during differentiation is essential. Here, we address this gap by providing a comprehensive analysis in both the HSPC and the myeloid progeny compartments in a humanized TET2-CH model. By studying the behavior of human TET2^Mut^ HSPCs under various stress conditions, our work provides valuable insights into the environmental factors that drive human TET2-CH. Remarkably, our findings also revealed an opposing effect of the TET2 mutation on specific cell types (or compartments, you choose) in the hematopoietic system, such that HSPCs show resilience in response to inflammation, but their myeloid progeny show an exacerbated inflammatory phenotype driven by the expansion of a hyperactivated monocyte subset.

## Results

### Loss of TET2 in human HSPCs confers growth advantage in LPS-induced stress

**Figure 1:**
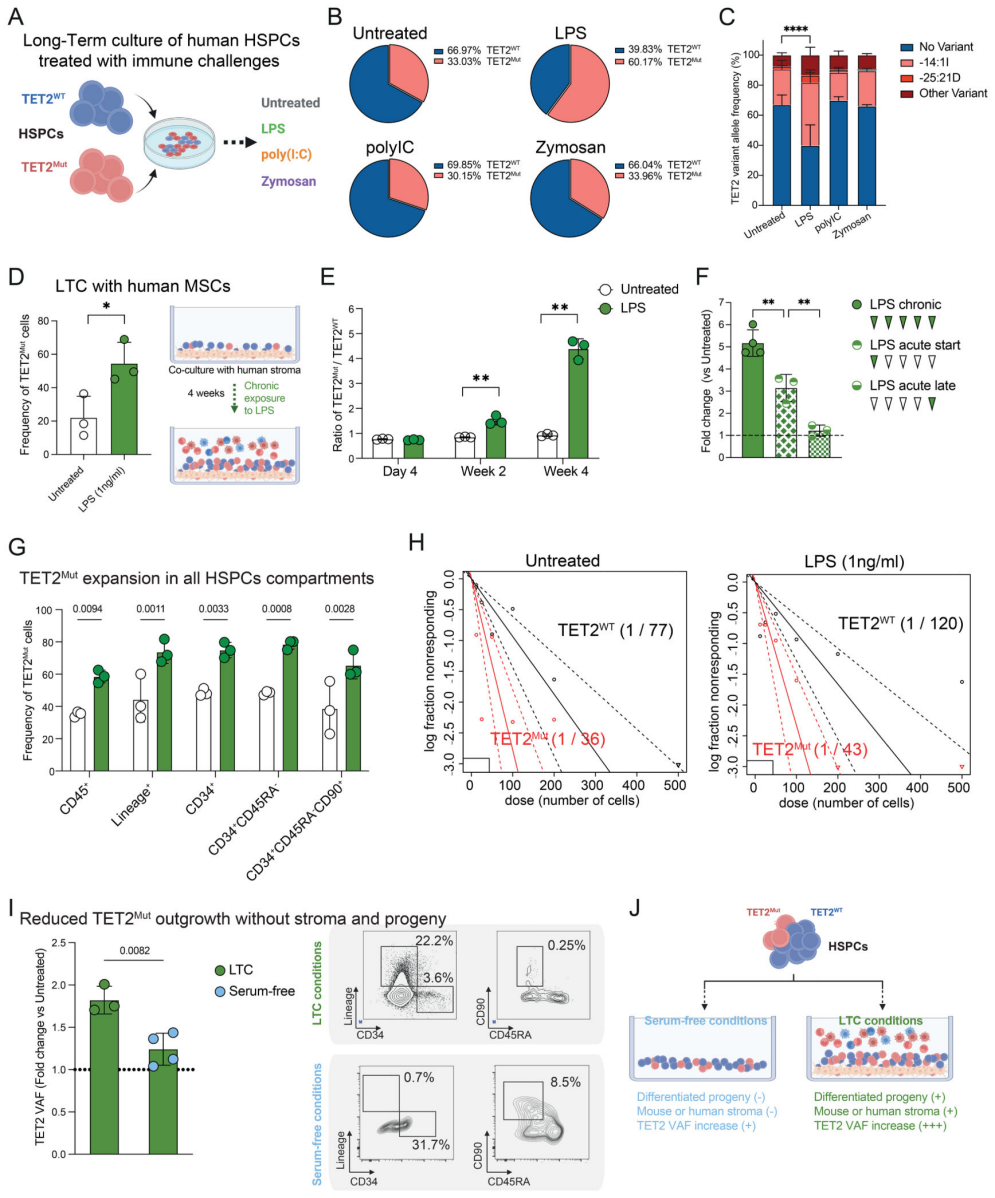
Myeloid progeny and stroma contribute to the growth advantage of TET2^Mut^ HSPCs in inflammatory environments. **A**. Schematic representation of the long-term culture (LTC) assay design to study the competitive fitness of TET2^Mut^ human HSPCs in different stress environments after immune challenges. Data in the figure is representative of at least three independent experiments using three different pools of biological donors. Created with BioRender. **B**. Frequency of the overall incidence of TET2 loss of function mutations at the end of the 5 weeks LTC competition assay. Percentage of TET2^WT^ or TET2^Mut^ genome reads from MiSeq are represented. Data shows the mean of three independent biological donors. **C**. Variant allele frequency (VAF) of the three most common variants; in blue, “no variant” means wild type variant, in red we can distinguish three types of TET2-mutant variants, being variant “-14:1” the most frequent (see Methods for details of CRISPR efficiency and variants engineered). Percentage of TET2^WT^ or TET2^Mut^ genome reads from MiSeq are represented. Data showing mean and SD from three biological replicates. Two-way ANOVA test used for significance, **** p<0.001. **D**. Frequency of the overall incidence of TET2 loss of function mutations at the end of 4 weeks LTC competition assay performed in co-culture with human mesenchymal stromal cells (MSCs). Each dot represents a technical replicate for the same experiment performed with a pool of different biological donors. Data showing mean and SD from three technical replicates. Unpaired t-test used for significance, * p<0.05. The supporting diagram was created with Biorender. **E**. Time kinetic of the cell ratio between CD45^+^HLA-A2^-^ (TET2^Mut^) and CD45^+^HLA-A2^+^ (TET2^WT^). Percentage of HLA-A2^-^ and HLA-A2^+^ cells were measured by flow cytometry (see Figure S1A) at day 4, day 14 and day 28 of the LTC competition assay. Each dot represents a technical replicate for the same experiment performed with a pool of different biological donors. Three independent experiments using three different pools of biological donors were performed obtaining similar results. Data showing mean and SD from three technical replicates. Two-way ANOVA test used for significance, * p<0.05; ** p<0.01. **F**. Fold change of the ratio between CD45^+^HLA-A2^-^ (TET2^Mut^) and CD45^+^HLA-A2^+^ (TET2^WT^) for each treatment condition compared to untreated condition. Each dot represents a technical replicate for the same experiment performed with a pool of different biological donors. Three independent experiments using three different pools of biological donors were performed obtaining similar results. Data showing mean and SD from three technical replicates. Two-way ANOVA test used for significance, * p<0.05; ** p<0.01. **G**. Ratio between TET2^WT^ and TET2^Mut^ cells for each cell subset compartment. Human CD45^+^, Lineage^+^, Lin^-^CD34^+^, Lin^-^CD34^+^CD38^-^ and Lin^-^CD34^+^CD38^-^CD45RA^-^CD90^+^ were analyzed. Each dot represents a technical replicate for the same experiment performed with a pool of different biological donors. Three independent experiments using three different pools of biological donors were performed obtaining similar results. Data showing mean and SD from three technical replicates. Two-way ANOVA test used for significance, p-values are shown in the figure. **H**. Long term culture initiating cell quantification from TET2^WT^ or TET2^Mut^ HSPC derived cultures in untreated or LPS (1ng/ml) treated conditions. **I**. Fold change of TET2 VAF and flow analysis showing differences between LTC and serum-free in vitro conditions (see Figure S1H). Time point for both conditions was 14 days. Each dot represents a technical replicate. Two independent experiments using different pools of biological donors were performed obtaining similar results. Unpaired t-test used for significance and p-value is shown in the figure. **J**. Schematic representation of the main differences observed between our two in vitro assays, the serum-free or the long-term culture media conditions. The supporting diagram was created with Biorender.

We previously characterized the model to study human TET2-CH by introducing *TET2* loss-of-function mutations in human HSPCs (22). We now aim to describe which immune insults trigger the selective advantage of TET2^Mut^ HSPCs. We performed long-term culture (LTC) of TET2^Mut^ human HSPCs in the presence of various pathogen-associated molecular patterns (PAMPs). We cultured TET2^Mut^ HSPCs in competition with TET2^WT^ HSPCs and challenged the system every week with lipopolysaccharide (LPS, bacteria), polyI:C (double strand RNA, virus), or Zymosan (fungi) to mimic different types of infection (Figure 1A). Interestingly, TET2-CH expand significantly after 4 weeks in the presence of LPS compared to other types of immune insults (Figure 1B-C), suggesting TLR4 and its downstream signaling factors as molecular targets of TET2 loss-of-function. From here, we decided to focus on LPS-mediated stress to characterize the cellular and molecular factors associated with the TET2-CH expansion. To gain insight into the advantage conferred by the TET2 mutation, we also performed non-competitive LTC assays to dissect the nature of the hematopoietic stress associated with LPS conditions (Figure S1A). Specific to LPS treatment, we observed an increase in myeloid differentiation from TET2^WT^-derived cultures. This is consistent with previous reports showing how LPS triggers robust myelopoiesis of HSPC compared to polyI:C (23,24). Of note, the myeloid expansion associated with LPS-stress was further exacerbated in TET2^Mut^ HSPCs (Figure S1B), consistent with our previous work reporting the myeloid bias of human TET2^Mut^ HSPCs in vivo (22). Further, TET2-CH expansion was independent of the source of stromal support for HSPCs, as both co-cultures with murine stroma cell line (MS5) or with primary human mesenchymal stromal cells (MSCs) show similar results (Figure 1D). Importantly, we also validated that the expansion of TET2-CH was independent of the origin of human HSPCs. We performed LTC during 20 days from CRISPR-edited human bone marrow CD34^+^ cells or HSPC derived from a wild-type or CRISPR-edited human iPSC clone. Across all *in vitro* models, we observed the outgrowth of TET2 variant allele frequency (VAF) in LPS-mediated stress (Figure S1C-D). To further characterize the cell subsets subjected to the increase of TET2 VAF in LPS-stress environments, we set up a competitive assay by mixing TET2^Mut^ with TET2^WT^ human HSPCs from different HLA types to quantify mutant cells by flow cytometry (Figure S1E). We pooled different cord blood donors of each HLA type and validated no significant difference between the HLA groups regarding the frequency of HSCs (defined as Lin^-^CD34^+^CD38^-^CD45RA^-^CD90^+^) and HSPCs, that could influence long-term outgrowth advantage (Figure S1F). In line with our initial observation, long-term competitive assays using the HLA-mismatch competition strategy revealed the TET2-CH outgrowth over time in LPS-stress environments (Figure 1E and S1G). Similar results were obtained following a single LPS challenge during the first week of the competitive assay and, not observed when LPS challenge was delayed to the week prior to experimental endpoint (Figure 1F). These findings suggest a causal relation between environmental conditions that drive myeloid expansion and TET2-CH outgrowth. At the end of the LTC assay, both differentiated progeny and more primitive HSPC subsets show an increase in TET2 VAF (Figure 1G). Consistently, using our long-term culture-initiating cell (LTC-IC) assay, the frequency of primitive HSCs upon LPS-stress environments is higher in TET2^Mut^ HSPC derived cultures (Figure 1H). To gain a deeper understanding of the contribution of differentiated progeny and the stroma to the expansion of TET2-CH, we also reproduced the competitive assay in *in vitro* conditions that support the expansion of undifferentiated hematopoietic stem and progenitor cells in the absence of stromal support (25,26). Thus, in the absence of both the stroma cell layer and differentiated progeny, the expansion of TET2-CH upon LPS-mediated stress was significantly reduced (Figure 1I-J and Figure S1H). Overall, this data suggests that TET2-CH outgrowth originates from a cell-intrinsic advantage of the TET2^Mut^ HSPC under LPS-mediated stress and this is further exacerbated by the presence of the myeloid progeny and the stroma.

### Cell-specific and opposite impact of TET2 loss in HSPCs and myeloid progeny

**Figure 2:**
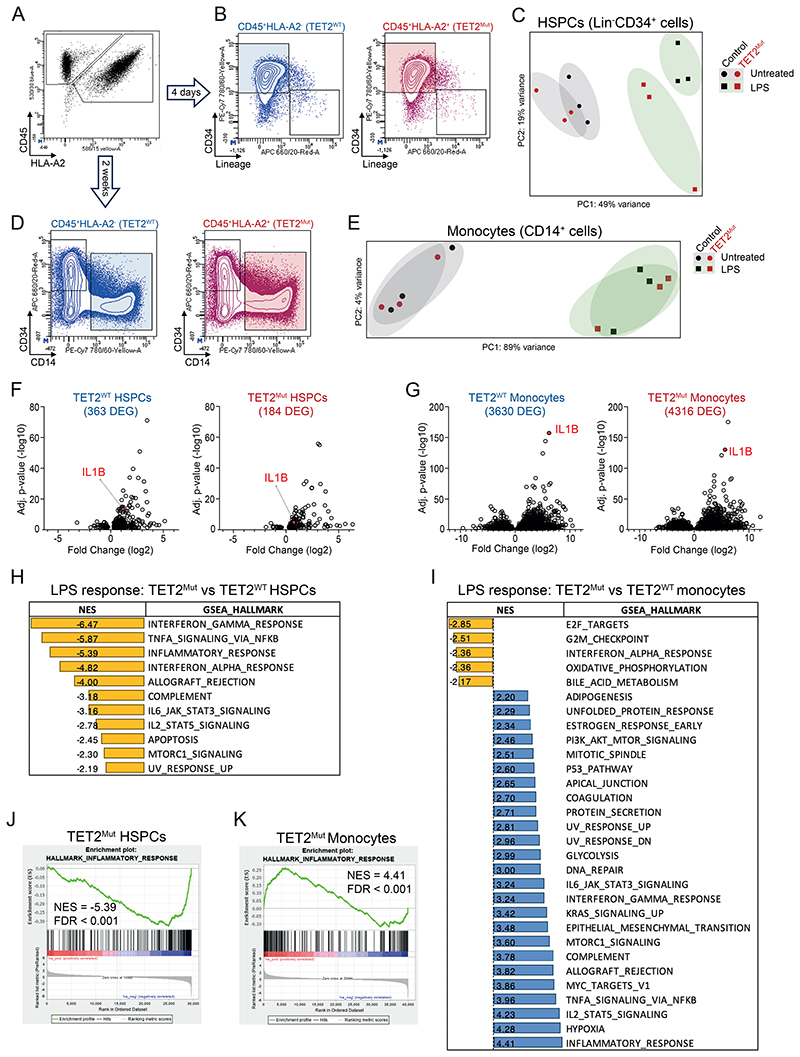
Loss of TET2 lead to opposite transcriptional responses in HSPCs and myeloid progeny upon LPS-mediated stress. **A**. Representative flow cytometry gating strategy to analyze HLA-based competition assays between TET2^WT^ and TET2^Mut^ human HSPCs. For each time point and cell type analyzed (CD34^+^ at day 4 and CD14^+^ at day 14) cells were sorted based on the HLA type to perform RNA-sequencing. **B**. Representative flow cytometry gating strategy to analyze HLA-based competition assays between TET2^WT^ and TET2^Mut^ human HSPCs at day 4 of the LTC assay to sort CD34^+^ cells from each HLA type. **C**. RNA-sequencing principal component analysis (PCA) of the different conditions (TET2^WT^ or TET2^Mut^ and Untreated or LPS) from CD34^+^ cell population analysis (n=3). **D**. Representative flow cytometry gating strategy to analyze HLA-based competition assays between TET2^WT^ and TET2^Mut^ human HSPCs at day 14 of the LTC assay to sort CD14+ cells from each HLA type. **E**. RNA-sequencing principal component analysis (PCA) of the different conditions (TET2^WT^/TET2^Mut^ and Untreated/LPS) from CD14^+^ cell population analysis (n=3). **F**. Volcano plot showing differentially expressed genes in TET2^WT^ (363 DEG, left panel) and TET2^Mut^ (184 DEG, right panel) HSPCs after LPS challenge. The dot corresponding to the IL1B gene is highlighted in red. See Supplementary Table 2 for list of genes. **G**. Volcano plot showing differentially expressed genes in TET2^WT^ (3630 DEG, left panel) and TET2^Mut^ (4316 DEG, right panel) monocytes after LPS challenge. See Supplementary Table 2 for list of genes. **H**. Gene Set Enrichment Analysis (GSEA) using Hallmark database for the comparison between TET2^WT^ CD34^+^ and TET2^Mut^ CD34^+^ upon LPS treatment (see Supplementary Table 3 for complete list of pathways and details). **I** GSEA using Hallmark database for the comparison between TET2^WT^ CD14^+^ and TET2^Mut^ CD14^+^ upon LPS treatment (see Supplementary Table 3 for complete list of pathways and details). **J**. GSEA showing downregulation of the inflammatory response pathway in TET2^Mut^ CD34^+^ compared with TET2^WT^ CD34+ in LPS-treated condition. **K**. GSEA showing upregulation of the inflammatory response pathway in TET2^Mut^ CD14^+^ compared with TET2^WT^ CD14^+^ in LPS-treated condition.

Intrigued by the opposite dynamics of TET2-mutant myeloid progeny (increased myeloid output) compared to HSPCs (attenuated LTC-IC exhaustion) in LPS-stress environments, we sought to compare the transcriptional response of both compartments (myeloid progeny vs stem/progenitor). We performed bulk RNA sequencing following our competitive assays, where TET2^WT^ and TET2^Mut^ cells were present within the same environment (Figure 2A). For the analysis of HSPCs, we isolated Lineage^-^CD34^+^ cells on day 4 of the LTC assay, 6 hours after one LPS challenge (Figure 2B-C). For the analysis of the myeloid compartment, we isolated monocytes (CD14^+^ cells) on day 14 of the LTC assay, also 6 hours after one LPS challenge (Figure 2D-E) to compare the impact of TET2 mutations on the transcriptional adaptation to stress between the two cell types. As expected, enrichment pathway analysis show that LPS treatment triggered the activation of the inflammatory response and associated transcriptional programs in both HSPCs and monocytes (Figure S2A-B and Supplementary Table 1). When comparing between the cell types at the transcriptional level, as specialized cells, monocytes elicit a greater robust transcriptional response to LPS challenge compared to HSPCs, as exemplified by upregulation of *IL1B* (Figure 2F-G and Supplementary Table 2). Surprisingly, when comparing the TET2^Mut^ cells to their wild-type counterparts, we observe an opposite phenotype in HSPCs than that seen in monocytes. In HSPCs, we observe that TET2^Mut^ cells are characterized by a decrease in differentially expressed genes, and the overall attenuation of transcriptional pathways and proinflammatory cytokines activated upon LPS (Figure 2F, H, and Supplementary Table 3). In contrast, TET2^Mut^ monocytes show an exacerbated increase of differentially expressed genes, and the hyperactivation of inflammatory response pathways and LPS-mediated transcriptional responses (Figure 2G, I and Supplementary Table 3). Our observation that human TET2^Mut^ myeloid cells show an exacerbated inflammatory response is in line with previous works (22,27). The cell-specific transcriptional signature seen in TET2^Mut^ HSPCs and monocytes results from the opposite trend of pro-inflammatory cytokines and chemokines expression between the two cell types (Figure 2J-K and Figure S2C-E). In particular, this was the case for target genes downstream of TLR4, the LPS receptor, and the *TLR4* gene itself. Indeed, we observe that TET2^Mut^ HSPCs downregulate the expression of TLR4 at the cell membrane (Figure S2F). To assess the direct influence of TLR4 activation on TET2-CH expansion, we reproduced our LTC assay in the presence of an anti-TLR4 antibody that blocks TLR4 signaling. We also included conditions with inhibitory molecules for downstream factors of TLR4, such as the JAK1/JAK2 inhibitor Ruxolitinib and the inflammasome/NLRP3 inhibitor MCC950 (28–31). TLR4 blockage or JAK1/JAK2 inhibition restored the TET2 VAF levels to those observed in untreated conditions (Figure S2G). The inflammasome inhibition attenuated the outgrowth of mutant cells (Figure S2G), likely by targeting the exacerbated production of IL1B by TET2^Mut^ myeloid progeny (11,32). Overall, our data identifies an opposing transcriptional adaptation of TET2^Mut^ human HSPCs to TET2^Mut^ myeloid progeny to an inflammatory environment.

### Human TET2-derived CH expands in environments triggering myelopoiesis

**Figure 3:**
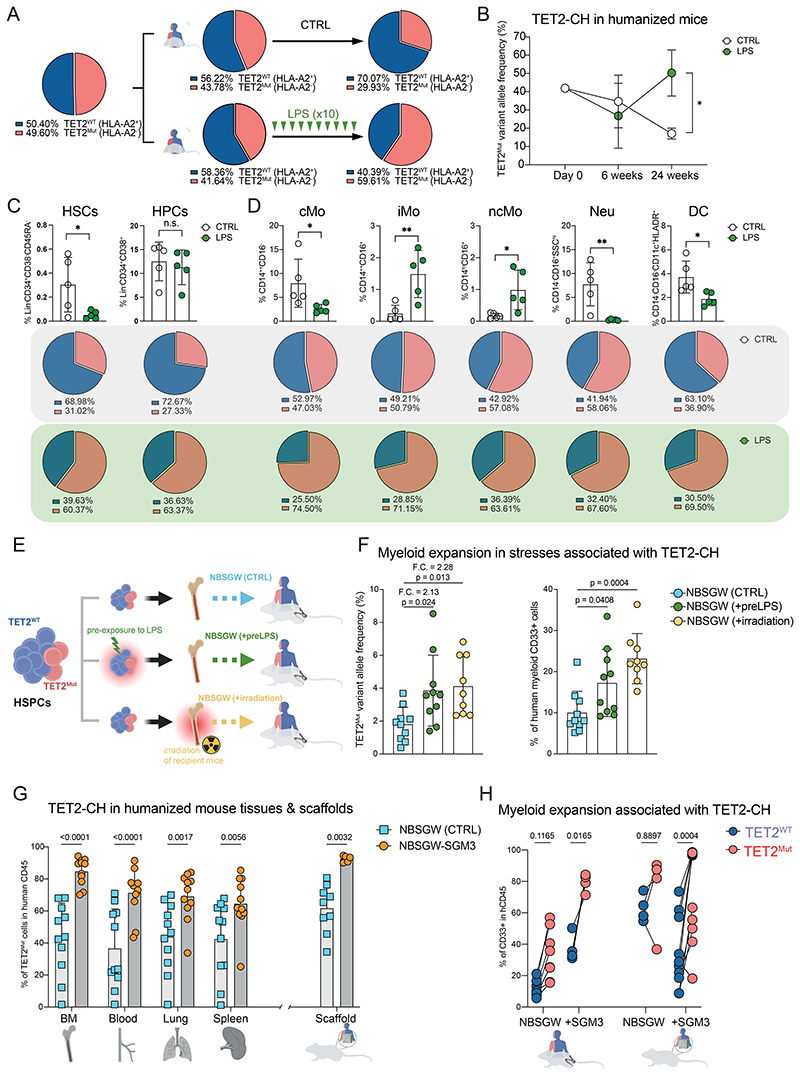
In vivo expansion of human TET2-CH in environments triggering emergency myelopoiesis. **A**. Schematic representation of the analysis of TET2-CH in the human hematopoietic system reconstituted overtime and after periodic intraperitoneal administration of LPS. Pie charts showing percentage of CD45^+^ HLA-A2^-/+^ for each group of mice (n = 5) before starting the LPS treatment at 6 weeks, and at the end of the experiment, at 24 weeks after the initial injection of human HSPCs. Human hematopoietic HLA-A2^-^ cells are derived from TET2^Mut^ HSPCs. Data showing mean from five humanized mice in each group. **B**. Quantification of TET2^Mut^-derived clonal hematopoiesis by showing variant allele frequency overtime of TET2 mutations in human CD45^+^ cells from bone marrow of mice. Data showing mean and SD from five humanized mice in each group. Two-way ANOVA test used for significance, * p<0.05. **C**. Quantification of hematopoietic stem (Lin^-^CD34^+^CD38^-^CD45RA^-^) and progenitor (Lin^-^CD34^+^CD38^+^) cells in untreated and LPS-treated humanized mice and the frequency of TET2^Mut^ cells within each cell subset displayed in pie charts. Data showing mean and SD from five humanized mice in each group, each dot represents one mouse. Unpaired t test used for significance; * p<0.05; n.s. p>0.05. **D**. Quantification of different myeloid cell subsets in untreated and LPS-treated humanized mice and the frequency of TET2^Mut^ cells within each myeloid subset displayed in pie charts. Data showing mean and SD from five humanized mice in each group, each dot represents one mouse. Unpaired t test used for significance; * p<0.05; ** p<0.01. **E**. Schematic representation of TET2-CH in vivo models to analyze different stress conditions. Along with the control (CTRL) group, we analyzed the impact of LPS solely on HSPCs (+ pre-LPS) before injecting the cells in the mice and the impact of bone marrow niche inflammation (+ irradiation) in the human engraftment long-term. **F**. Variant allele frequency (VAF) of TET2 in human CD45+ (left panel) and percentage of human myeloid cells (right panel) in the three experimental groups of humanized mice, 16 weeks after the injection of HSPCs. Data showing mean and SD from 9-10 humanized mice in each group, each dot represents one mouse. Data from two independent experiments. Two-way ANOVA test used for significance, p-values are shown in the figure. F.C., fold change compared to CTRL group. **G**. Quantification of TET2^Mut^-derived clonal hematopoiesis in the different tissues and scaffolds by showing percentage of CD45^+^ human cells derived from TET2^Mut^ HSPCs. (See Figure S4C-D). Same mix of TET2^WT^ and TET2^Mut^ HSPCs in competition was injected in both NBSGW and NBSGW-SGM3 to compare the dynamic of TET2-CH. Data showing mean and SD from two independent experiments, each dot represents one mouse. Two-way ANOVA test used for significance; p-values are represented in the figure. **H**. Quantification and comparison of myeloid progeny derived from TET2^WT^ HSPCs or TET2^Mut^ HSPCs in the same humanized mouse. Same mix of TET2^WT^ and TET2^Mut^ HSPCs in competition was injected in both NBSGW and NBSGW-SGM3 to compare the myeloid expansion between TET2^WT^ and TET2^Mut^-derived hematopoietic system. Data showing mean and SD from two independent experiments, each dot represents one mouse. Paired t-test used for significance; p-values are represented in the figure.

We next sought to recapitulate the human TET2-CH expansion *in vivo* with our humanized mouse model (22). First, we analyzed the engraftment capacity and lineage output of TET2^Mut^ human HSPCs in NBSGW mice (Figure S3A-B). TET2^Mut^ HSPCs show no difference in the level of engraftment but have a myeloid bias, consistent with observations in *TET2*-mutated individuals (33) (Figure S3C-D). We also analyzed the competitive fitness of TET2^Mut^ HSPCs by injecting both TET2^WT^ and TET2^Mut^ HSPCs in NBSGW mice. We did not find significant outgrowth of TET2-CH in primary or secondary recipient mice (Figure S3E-F). To mimic the conditions where we observed TET2-CH outgrowth in our *in vitro* models, we subjected TET2-CH humanized mice to a regimen of LPS injections. This *in vivo* model recreated a sustained systemic inflammation, as exemplified by the detection of human proinflammatory cytokines (Figure S3G). In this environment, we observed a robust human TET2-CH outgrowth (Figure 3A-B). Indeed, the human hematopoietic system of the LPS-treated mice show the repercussions of the inflamed environment in the bone marrow. The frequency of HSPCs was severely reduced and this correlated with the increase of the inflammatory intermediate and non-classical monocytes in the bone marrow (Figure 3C-D and Figure S3H-I). Consistent with the *in vitro* model, the increase of mutant cells was present in both the stem and precursor compartments, as well as in the myeloid subsets analyzed in the bone marrow (Figure 3C-D).

Our results showing no significant advantage of human TET2^Mut^ HSPCs in the absence of exogenous stress differs from previously reported works, whereby it is described that mouse TET2-KO HSPCs have an advantage when transplanted in irradiated mice (34,35). One possible explanation points to irradiation, as it is a stressor that could favor TET2-CH by creating an inflammatory environment in the bone marrow via tissue damage and derepression of transposable elements (36,37). Thus, we sought to determine the impact of preconditioning the niche (irradiation) in our humanized TET2-CH model. In contrast, we compared this to the influence of preconditioning and activating solely HSPCs (without activating the myeloid progeny) with LPS *ex vivo* (pre-LPS) and assessing the expansion of TET2-CH *in vivo*. (Figure 3E). Irradiation of the NBSGW mice increases the engraftment of human cells in the bone marrow, while the *ex vivo* challenges of HSPC with LPS, before the injection in the mice, slightly reduces their engraftment capacity (Figure S4A). Interestingly, both stresses shape the human engraftment by increasing the TET2 VAF and triggering human myelopoiesis (Figure 3F and Figure S4B).

Intrigued by the association between TET2-CH outgrowth and emergency myelopoiesis, we sought to determine whether TET2^Mut^ clones would expand in a context of active human myelopoiesis independently of PAMPs (LPS) that trigger immune response and the presence of classical inflammatory cytokines. We took advantage of the humanized NBSGW-SGM3 mouse model, which produces human GM-CSF, SCF and IL-3, that pushes HSPCs to produce robust systemic human myelopoiesis in different organs (Figure S4C). Compared to NBSGW mice, we detected a robust increase of TET2-CH in different humanized organs of NBSGW-SGM3 mice (Figure 3G and Figure S4D). Moreover, we also analyzed the TET2-CH in our *in vivo* humanized scaffold model to assess whether a human stroma niche could influence the TET2-CH growth dynamic (38). Implantation of the scaffold was done after pre-seeding the mix of human TET2^WT^ and TET2^Mut^ HSPCs (ratio 1:1) with the MSCs into the scaffold, allowing us to account for possible homing of HSPCs to the mouse bone marrow. Interestingly, we observe a clear TET2-CH expansion in the scaffolds implanted in NBSGW-SGM3, but not in the scaffolds implanted in NBSGW (Figure 3G). Of note, in both bone marrow and scaffold models, we observe a significant correlation between myeloid expansion driven by the SGM3 background and TET2-CH outgrowth (Figure 3H). We then injected intravenously the mix of TET2^WT^ and TET2^Mut^ HSPCs (ratio 1:1) into mice harboring a humanized scaffold pre-seeded only with human MSCs to analyze the influence of the mutation on homing to the different niches (i.e. human in the scaffold and mouse in the bone marrow). Although the human engraftment was low in the scaffolds, we observe similar TET2-CH incidence between the bone marrow and the scaffolds of the same mouse (Figure S4E-F). Collectively, our results illustrate how environments that lead to active myelopoiesis (i.e. systemic inflammation or SGM3 environment) drive the outgrowth of human TET2-derived CH (Figure S4G).

### Human TET2^Mut^ HSCs show dampened activation upon systemic inflammation

**Figure 4:**
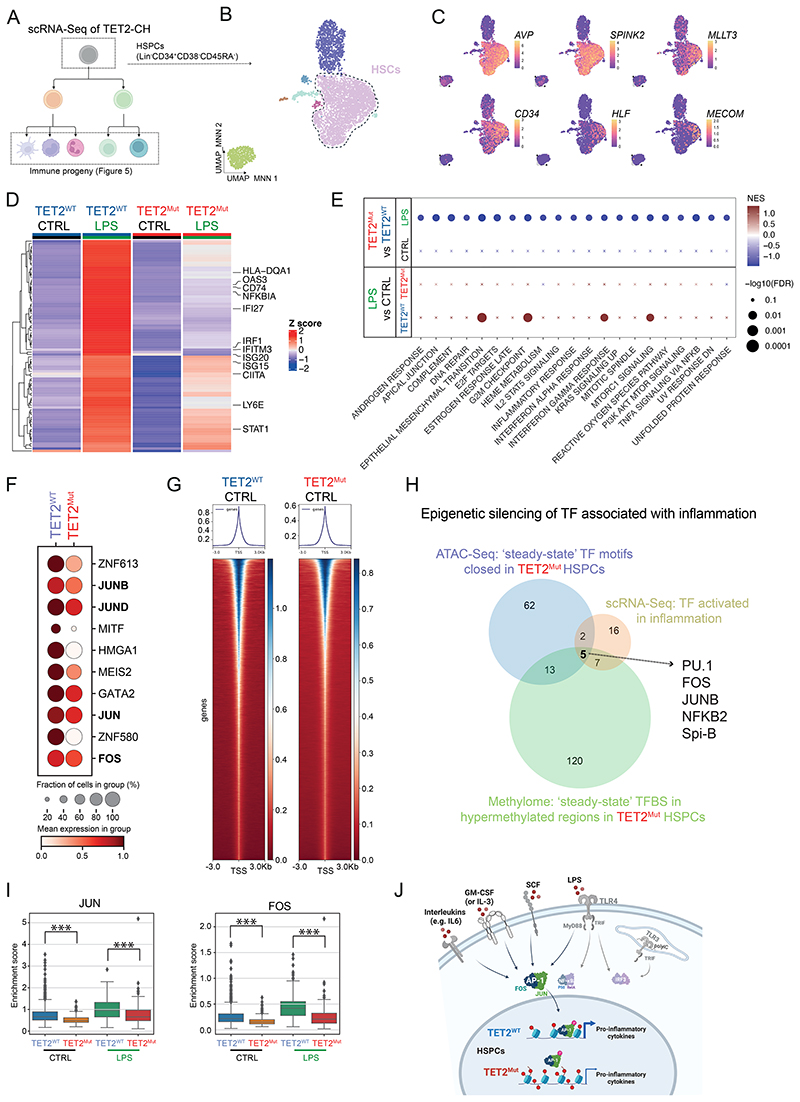
TET2^Mut^ HSPCs show an attenuated transcriptional response to active inflammation by epigenetic silencing of JUN and FOS. **A**. Schematic representations of the single cell RNA-Sequencing analysis both at the HSPC (see Figure 4B-E) and immune progeny (see Figure 5) compartment. The figure was created with Biorender. **B**. Integrated uniform manifold approximation and projection (UMAP) for dimension reduction representation of human CD45^+^Lineage^-^CD34^+^CD38^-^CD45RA^-^ cells (see Figure S4A for gating strategy) sorted from humanized mice from Figure 3A. **C**. UMAPs representing expression of canonical genes associated with stemness. **D**. Heatmap representing differentially expressed genes between untreated and LPS-treated conditions within the TET2^WT^ HSC cluster. Each column corresponds to the HSC cluster for one of the four conditions, and each row shows the scaled mean normalized expression of a gene. **E**. Bubble plot showing the GSEA using Hallmark database for the different comparisons displayed in the row header. Only transcriptional pathways that were significant for one of the comparisons are displayed in the figure. Transcriptional pathways with no significant enrichment are represented with a cross. **F**. Top 10 transcription factors differentially activated at steady-state (CTRL) between TET2^WT^ (blue) and TET2^Mut^ (red) HSCs. **G**. Heatmap representing the open chromatin signal in TET2^WT^ (left panel) or TET2^Mut^ (right panel) HSPCs surrounding 3 kilobases from the transcription start site (TSS). ATAC-Seq was performed from human HSPC (CD45^+^Lin^-^CD34^+^CD38^-^CD45RA^-^ cells) isolated from humanized mice after 16 weeks from the HSPC injection. Figure shows a representative example for TET2^WT^ HSPCs (n = 4) and TET2^Mut^ HSPCs (n = 7). **H**. Venn diagram representing the cross-comparison of the three datasets (scRNA-Seq, methylome and ATAC-Seq) revealing that 5 transcription factors activated upon inflammation (see supplementary Table 4) have their binding sites located in regions that are hypermethylated (see Supplementary Table 5) and less accessible (see Supplementary Table 6) in TET2^Mut^ HSPC. **I**. Enrichment score for the expression of JUN (left panel) and FOS (right panel) target genes in the HSC cluster from our in vivo scRNA-Seq (see Figure 4A). Two-sided independent t-test was used for significance; *** p<0.001. **J**. Schematic representation of the signaling pathways converging in AP-1. The figure summarizes the different receptors triggering JUN and FOS activation that are related with our in vivo models showing TET2-CH expansion. The figure was created with Biorender.

Finally, we performed single cell RNA-sequencing (scRNASeq) to gain insight into the specific cell types that drive the opposing effects at both the HSPC and immune progeny compartments from our humanized mice that were subjected to serial challenges of LPS (Figure 4A). To characterize the response to inflammation of TET2^Mut^ HSPCs *in vivo* we performed FACS and scRNASeq analysis of human Lin^-^ CD45^+^CD34^+^CD38^-^CD45RA^-^ cells from the humanized mice. Both the uniform manifold approximation and projection (UMAP) embedding and unbiased clustering of sorted cells from untreated and LPS-treated mice identified a clear cluster of cells expressing a robust HSC signature (Figure 4B-C). We focused our downstream analysis on the transcriptional response to inflammatory stress on the HSC cluster. We then identified the differentially expressed genes associated to LPS-stress in human HSCs to evaluate the impact of the mutation. The heatmap representing all 4 conditions reveals that the genes activated in TET2^WT^ HSCs by an inflammatory environment are dampened in TET2^Mut^ HSCs exposed to the same environment (Figure 4D). Next, we used gene set enrichment analysis to demonstrate that while TET2^Mut^ HSC show no significant pathways altered by the inflammatory environment (LPS vs CTRL), TET2^WT^ HSCs significantly upregulate pathways associated with cell cycle, active cell growth and metabolism, and LPS-mediated stress response (Figure 4E and Figure S5A). At the same time, we show that the activation of transcriptional pathways in HSC from LPS-treated mice was significantly downregulated in TET2^Mut^ HSCs (Figure 4E). Next, we focused our analysis on the transcription factors (TFs) responsible for mounting the inflammatory response in the HSC cluster. As expected, PU.1, STAT1, FOS and JUN appear as the TFs that are more active in TET2^WT^ HSPCs from LPS-treated mice (Figure S5B and Table S4). We observe that the activity of these TFs is reduced in TET2^Mut^ HSC from LPS-treated mice, consistent with the downregulation observed in their downstream transcriptional programs (Figure S5B and Table S4). Interestingly, when we analyze the activity of TFs between TET2^WT^ and TET2^Mut^ at steady-state, JUN and FOS also appear with reduced activity in TET2^Mut^ HSCs (Figure 4F). To further characterize the cell-specific impact of TET2 loss of function on HSCs, we performed methylome and transposase-accessible chromatin sequencing (ATAC-Seq). TET2^Mut^ human HSCs show an overall DNA hypermethylation status and the ATACSeq revealed an overall closed chromatin status around transcription start sites (TSS) (Figure 4G and Figure S5C). To better understand whether steady-state TET2^Mut^ HSCs are intrinsically prepared to respond differently to a future stress, we cross-compared the list of TFs activated in HSCs during an inflammatory environment (Table S4) with the list of TFs motifs present in hypermethylated and closed chromatin regions (Table S5 and Table S6). Interestingly, when we cross-compared the three datasets, we reveal a slight overlap involving just five TFs: FOS, JUN and NFKB2, which are involved in the immediate signaling pathway downstream of TLR4, activating the expression of inflammatory cytokines, and PU.1 and Spi-B, which bind identical DNA motifs and are involved in the differentiation of HSCs (Figure 4H). Integrated multi-omics profiling from our *in vivo* model, demonstrates that TET2^Mut^ human HSCs, exhibit epigenetic silencing of AP-1 constituents FOS and JUN, conferring a cell-autonomous resistance to activation by both innate immune insults (LPS/TLR4) and hematopoietic cytokine/growth factor inputs (GM-CSF, SCF, IL-3) that physiologically converge upon AP-1–driven transcriptional programs (39–43) (Figure 4I-J).

### Hyperreactive monocytes and distinct macrophage trajectory in TET2-CH

**Figure 5:**
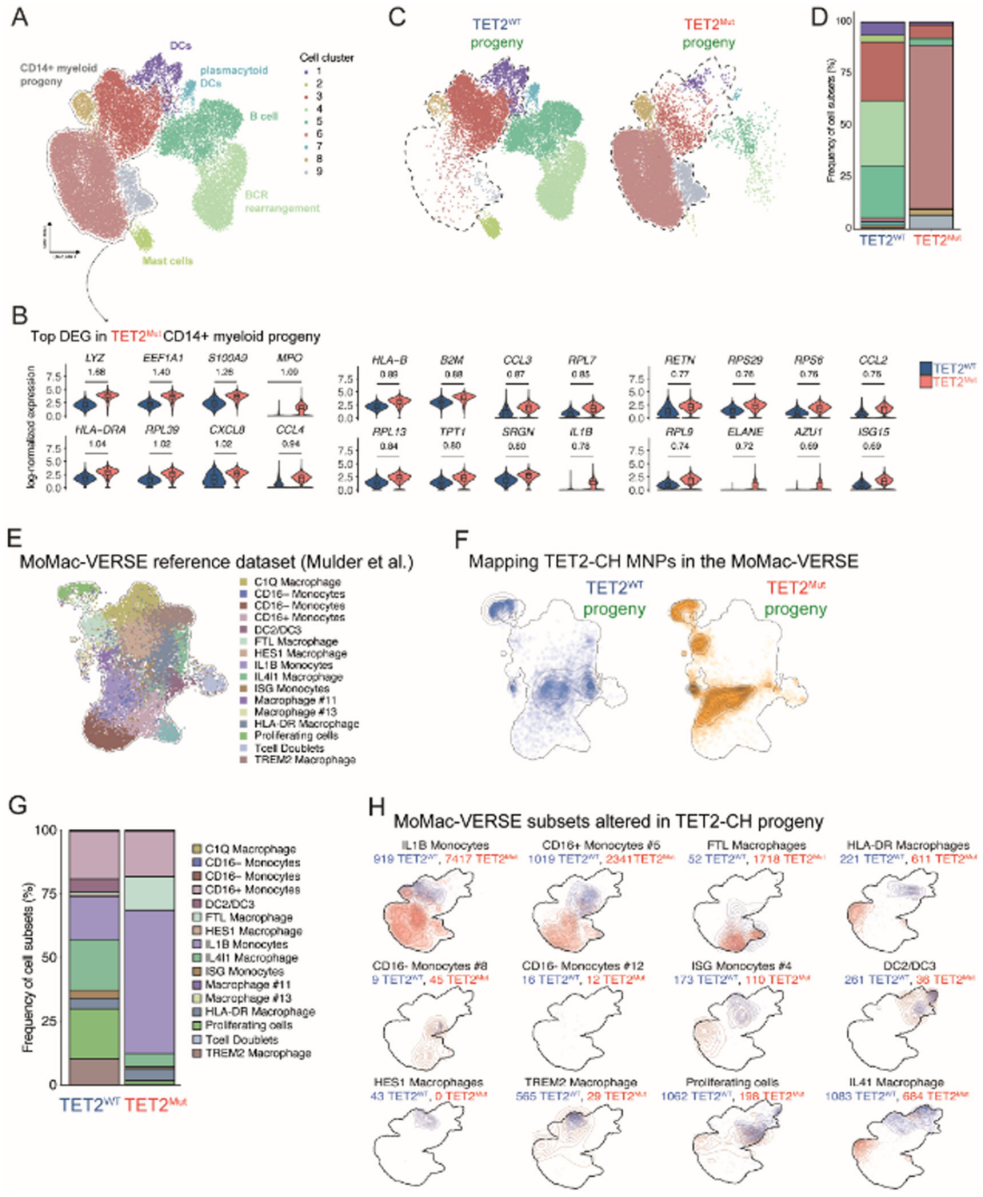
Immune progeny derived from TET2^Mut^ HSPCs is composed by an expansion of hyperactivated monocytes with a distinct macrophage differentiation trajectory. **A**. UMAP for dimension reduction analysis of the immune progeny derived in LPS-treated mice (see Figure S5A for gating strategy). Cell cluster annotation was done based on the expression of canonical genes associated with cell lineages (see Figure S5B-C). **B**. Violin plots of the top 24 differentially expressed genes between CD14^+^ cells isolated from immune progeny (see Figure S5C) derived from TET2^WT^ (blue) or TET2^Mut^ (red) HSPCs. Log2 fold change is displayed in the figure. **C**. UMAP for dimension reduction analysis separating immune progeny derived from TET2^WT^ or TET2^Mut^ HSPCs. Dashed line delineates mononuclear phagocyte cell subsets (see Figure 4J). **D**. Quantification in stacked bar chart of the different cell subsets derived from TET2^WT^ or TET2^Mut^ HSPCs. **E**. UMAP for dimension reduction representation of MoMac-VERSE reference dataset characterized previously by Mulder et al.(45) **F**. Projection of mononuclear phagocytes derived from TET2^WT^ or TET2^Mut^ HSPCs into the Mo-Mac VERSE **G**. Quantification in stacked bar chart of the different cell subsets, derived from TET2^WT^ or TET2^Mut^ HSPCs, defined based on the MoMac-VERSE cell signatures. **H**. Distribution in UMAP embedding of TET2^WT^ (blue) or TET2^Mut^ (red) derived progeny grouped by MoMac-VERSE transferred label. Cell numbers from TET2^WT^ or TET2^Mut^ progeny corresponding to each cell type signature are displayed in the figure.

**Figure 6:**
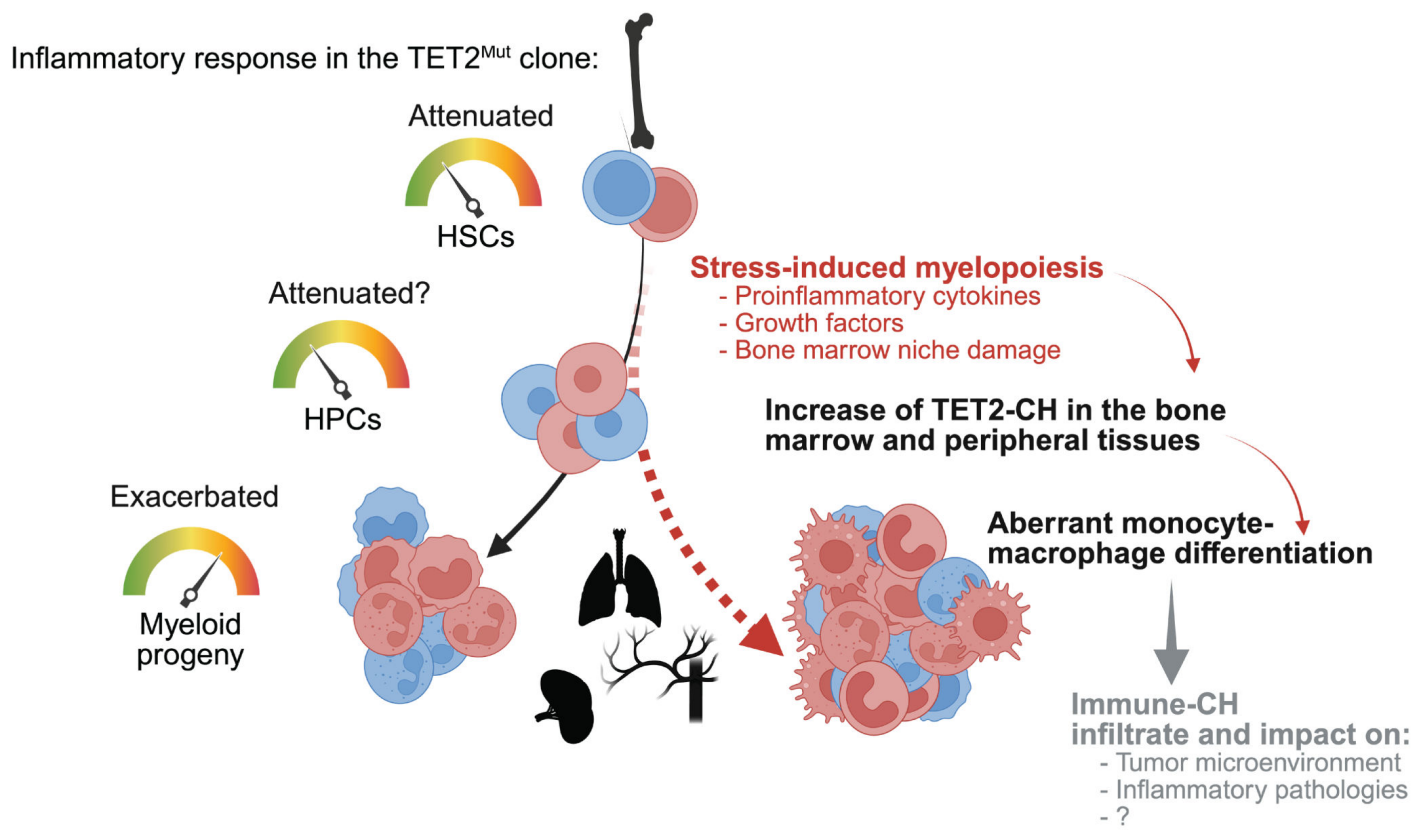
Aberrant monocyte-macrophage differentiation during stress-induced myelopoiesis drive human TET2-mutant clonal hematopoiesis Schematic representation of the opposite impact of TET2 loss on HSPC and myeloid progeny, upon environmental stress. Environments triggering emergency myelopoiesis expand TET2-CH in tissues leading to the development of a distinct monocyte-macrophage trajectory that can drive pathological scenarios.

Lastly, we sought to also provide a single-cell resolution insight of the opposing effect associated to TET2 mutation at the myeloid cell level (Figure S6A). We first integrated the human cell progenies sorted from humanized mice reconstituted with either TET2^WT^ or TET2^Mut^ HSPCs into our analysis to avoid the potential confounding effect in the wild-type progenies cohabiting with TET2^Mut^ cells in our TET2-CH model used previously. We obtained a representative human immune cell repertoire reconstituted in NBSGW mice, identifying B cells, mast cells, dendritic cells (DCs), plasmacytoid dendritic cells (pDCs) and 4 major subsets of CD14^+^ myeloid cells (Figure 5A and Figure S6B-C). To confirm our *in vitro* data from differentiated myeloid cells, we first analyzed the impact of TET2 mutations in all CD14^+^ cells. The top differentially upregulated genes in TET2^Mut^ cells were composed of inflammatory cytokines and chemokines (Figure 5B), which aligns with our *in vitro* data and further reinforce the opposing response observed in HSPCs. When analyzing the lineage distribution, we noticed an overall myeloid bias with a decreased in mast cell differentiation derived from TET2^Mut^ human HSPC, which is consistent with previous reports (33,44) (Figure 5C). Moreover, when analyzing the specific cell subset distribution within the mononuclear phagocytes (MNPs), we distinguish a clear imbalance: DCs and CD14 cluster #3 subsets are enriched in progeny derived from TET2^WT^ HSPCs while CD14 clusters #6, #8 and #9 are enriched in TET2^Mut^ HSPC progeny (Figure 5C-D). When comparing the expression of inflammatory cytokines and chemokines between the two mains MNP CD14 clusters (#3 and #6), we observe an exacerbated activation associated with the cluster #6, which suggests that the hyperinflammation associated with TET2-derived CH is driven by specific myeloid subsets differentiated from TET2^Mut^ HSPC (Figure S6D). To better define the pathogenic inflammatory states acquired by TET2^Mut^ MNPs, we used MoMac-VERSE, a scRNASeq compendium that provides the conserved signatures of monocytes and macrophages across tissues (45) (Figure 5E). Conveniently, by using MoMac-VERSE, we distinguish the different cell subsets within the MNP progenies (Figure S7A) and which of these cell subsets are associated with different types of cancer or inflammatory disorders such as COVID or colitis (Figure S7B-C). When we mapped the TET2^WT^ and TET2^Mut^ MNPs into MoMac-VERSE, we observed that they occupied distinct areas (Figure 5F). The TET2^WT^ myeloid progeny is characterized by a repertoire of different monocytes and macrophages states, while the TET2^Mut^ progenies were characterized by the expansion of IL1B-monocytes and FTL (ferritin light chain)-macrophages (Figure 5G). These are MoMac subsets previously associated with inflammatory disorders such as osteoarthritis and rheumatoid arthritis (45). Consistently, when we projected the different MoMac-VERSE subsets into our humanized MNP dataset, we observed that IL1B monocytes and FTL macrophages mapped into areas dominated by TET2^Mut^ progenies (Figure 5H). Conversely, anti-inflammatory IL4I1 and TREM2 macrophages states mapped into areas dominated by TET2^WT^ cells (Figure 5H). IL4I1 and TREM2 macrophage signatures were found in macrophages of the tumor microenvironment (45) (Figure S7B-C). Altogether, these findings indicate that the myeloid progenies derived from TET2^Mut^ HSPCs are not only composed by hyper-activated monocytes but also likely produce distinct trajectories of monocyte-macrophage differentiation that could eventually shape the host response in the context of the solid tumor microenvironment (Figure 6).

## Discussion

This work contributes to a deeper understanding of how environmental cues shape the landscape of human TET2-derived clonal hematopoiesis. We link the selective advantage of TET2^Mut^ HSPCs to specific episodes of inflammation that drive emergency myelopoiesis. Even in our model of “sterile” emergency myelopoiesis, when injecting human HSPCs in the NBSGW-SGM3 mice, we observe a robust expansion of TET2-CH. We believe the data from this model is of special interest, since we observed the outgrowth of TET2-CH in the absence of an active infection/pathogen agent and the corresponding production of classical proinflammatory cytokines IL6, TNFα or IL1β that are previously associated to favor TET2-CH in mouse models (14,18,46). This data places, beyond proinflammatory cytokines, emergency myelopoiesis as a main phenomenon driving clonal hematopoiesis. Along these lines, it is tempting to speculate about the repercussions of SCF, IL3 and/or GM-CSF in the selection of mutant clones. As our *in vivo* model revealed, environments rich in these factors may favor TET2^Mut^ human HSPC and thus, clinical practices or *in vitro* protocols that use growth factors may account for the introduction of clonal selection (25,30,47).

The epigenetic silencing of FOS and JUN observed at steady-state, provides a unifying explanation for how diverse environmental stress that elicit emergency myelopoiesis selectively favor TET2-CH through a shared mechanism. In this context, FOS and JUN, as prototypical AP-1 transcription factors, are activated downstream of TLR4 (LPS), c-KIT (SCF), IL-3 and GM-CSF receptor pathways. In contrast, the immune signaling cascade elicited by polyI:C (TLR3), only marginally engages AP-1 while driving robust IRF activation and type I interferon production, consistent with our observation of TET2-CH expansion is only significant under LPS stimulation. Beyond the cell-autonomous mechanism, our data also reveals that the activation of myeloid progeny and bone marrow stroma in inflammatory environments, exacerbate the outgrowth of TET2-CH. Future work is needed to address the connection between the “hyporesponsive” TET2^Mut^ HSPC and the “hyperresponsive” TET2^Mut^ monocytes and to determine how the response from the bone marrow microenvironment to different types of stress influences the progression of clonal hematopoiesis.

Our work also highlights certain discrepancies between mouse and human TET2-CH models. In contrast to previous reports, showing that the HSPC compartment of TET2-KO models also upregulates proinflammatory cytokines such as IL6 (48), our work in the context of human cells, shows an opposite behavior, whereby human HSPCs are characterized by a mitigated upregulation of inflammation both *in vitro* and *in vivo*. Along these lines, a previous report has shown that mutant HSPCs from individuals carrying TET2 mutations are characterized by a mild activation upon inflammatory stress (49). Another relevant difference between our TET2-CH humanized mouse model and the classical TET2-KO mouse models is that we did not observe a significant outgrowth of the TET2 mutant clones when we transplanted the TET2^Mut^ HSPC in the absence of additional stress (NBSGW mice). Our data showing the increase of TET2 VAF in irradiated NBSGW mice compared to non-conditioned NBSGW mice points to irradiation of recipient mice as a possible factor that contributes to TET2-CH expansion in bone marrow transplant experiments. Therefore, to avoid confounding effects, working with experimental models that maintain a native bone marrow microenvironment is advised when assessing environmental cues that could drive clonal hematopoiesis (18,50).

A relevant idea that emerged from our work is the analysis of CH across different tissues, including non-hematopoietic tissues such as the lung. Considering the infiltration and presence of TET2-CH progeny in the lung, and our analysis revealing the distinct monocyte-macrophage differentiation trajectories, it is tempting to speculate that variations in tissue-resident immune cell composition in CH individuals, might influence the immune response in the context of other diseases. In particular, the role of CH progeny in the solid tumor microenvironment (51,52). Indeed, the human myeloid progeny from the lung of our humanized mouse model engrafted with TET2^Mut^ HSPC is less capable of controlling the growth of tumor organoids in vitro (53). In mouse models, loss of TET2 in myeloid progeny slowed melanoma growth by increased expression of proinflammatory M1 signatures and decreased expression of immunosuppressive M2 signatures (54). However, in humans, CH has been associated with worse survival in individuals following anti-PD-1/L1 therapy to different types of cancer (55). In this context, our analysis of the myeloid progenies differentiated from TET2^Mut^ human HSPCs captures such complexity. TET2^Mut^ myeloid progenies are both characterized by an exacerbated expansion of inflammatory monocytes that could fuel and contribute to tumorigenicity but also by reduced tumor-associated macrophages (TAMs) states differentiated from TET2^Mut^ monocytes (so less tumorigenic) (56). Overall, our work emphasizes the challenge of deciphering the complex and multifaceted interactions between specific CH mutations, the cell-specific impact of the mutation, and the specific environmental challenge they face.

## Methods

### Introduction of TET2 loss of function mutations in primary human HSPCs

Human HSPCs were isolated from umbilical cord blood (UCB) samples collected from full term donors after written informed consent at the Royal London Hospital (London, U.K.) or the Anthony Nolan cell therapy service. This study was conducted in accordance with the Declaration of Helsinki under the approval by the East London Ethical Research committee. HSPCs were isolated as described previously (57). In brief, Mononuclear cells (MNCs) were isolated by density centrifugation using Ficoll-Paque (GE 67 Healthcare) then depleted for lineage marker positive cells using an EasySep Human Progenitor Cell Enrichment Kit (Stem Cell Technologies). After sorting, HSPCs (Lin-CD34+CD38-) were seeded in StemSpanSFEM (Stem Cell Technologies) supplemented with 100 ng/mL rhFLT-3L, 100 ng/mL rHSPCF and 100 ng/mL rhTPO (Thermofisher-Peprotech, Cat# 300-19-10UG, 300-07-10UG and 300-18-10UG respectively). Introduction of TET2 loss of function mutation by CRISPR was done following our previous protocol (22). Human HSPCs were collected for CRISPR editing after 48 hours from the cell sorting. For CRISPR editing we used the NEON Transfection system (Thermofisher). After the electroporation, cells were cultured in the same media for 48 hours before being used in our ex vivo or in vivo assays. CRISPR efficiency was analyzed by next generation sequencing. All *in vivo* experiments analyzed, included humanized mice with >60% efficiency in human hematopoietic system reconstitution in the bone marrow. As previously reported (22), in all our experiments CRISPR efficiency was >90% of TET2 mutations that cause premature stop in exon 11 (from Y1576 the insertion of an A base pair in the DNA sequence leads to 7aa and a stop codon) and is therefore likely to be degraded via the NMD pathway or generate a truncated protein that lacks key residues in the catalytic domain.

### Long-term culture assay of TET2^Mut^ human HSPCs in the presence of immune challenges

For long term culture (LTC), first we expanded the MS-5 feeder cell layer. 2 days before initiating the LTC, MS-5 cells (RRID:CVCL_2128) were plated in 6-well plates (Falcon) previously coated with type-I collagen. Once they reached 70-80% confluency MS-5 were irradiated at 7.5 Gy and 6 to 18 hours later, the culture media was exchanged to Myelocult H5100 (Stemcells Technologies), being at least a day before seeding the HSPCs. On day 0 of the LTC, HSPCs were plated at 2-3 × 10^3^ cells/well. Cells were cultured at 37 °C in 5% CO_2_-humidified incubators. On day 2 of the LTC, cells were treated with the different PAMPs and immune challenges: for LPS we used 1μg/ml and 1ng/ml as specified in the figure legends, for polyI:C we used 5μg/ml, for Zymosan we used 5μg/ml. Every week, half medium changes were performed with the addition of new immune challenges. After 4 weeks, cells were collected and analyzed by flow cytometry and/or pelleted for DNA extraction and sequencing to assess the variant allele frequency (VAF) of TET2 mutations. For LTC assays followed by limiting dilution assay (LDA) human cells from the LTC were magnetically separated using mouse Sca1 positive selection kit (Stem Cell Technologies) according to the manufacturer’s instructions. Human cells were plated on a freshly prepared irradiated MS-5 layer in 96-well plates following our previous protocol (58). They were cultured, in normoxic conditions, with no addition of immune challenge for 2 weeks and positive wells were scored for the LTC-IC frequency score.

### Serum-free HSPC cultures

CRISPR-edited CD34+ cells were subjected to CRISPR gene targeting as above, and cultured in IMDM media (Gibco cat# 12440-053) supplemented with penicillin-streptomycin-glutamine (1X; Gibco cat# 10378-016), insulin-transferrin-selemiumethanolamide (1X; Gibco cat# 51500-056), Soluplus (0.1% w/v; BASF cat# 50539897), 740 Y-P (5 μM; Selleckchem cat# S7865), Butzyamide (100 nM; Selleckchem cat #E1352 ), UM729 (1.5 μM; StemCell Technologies cat# 72332), recombinant human FLT3L (10 ng/ml; Peprotech cat# AF-300-19), and monothioglyercol (100 μM; Sigma cat# M1753) (59). Media was changed every 48-72h, and in the LPS condition, supplemented with LPS at a concentration of 1μg/ml. After 10 or 14 days, cells were analyzed by FACS or pelleted for DNA extraction and sequencing.

### Generation of humanized mice reconstituted with control or TET2^Mut^ primary human HSPCs

In this work, we used “NBSGW” (NOD/SCID/ IL2rγ−/−/Tyr+/Kit W41J) mice, which were originally obtained from The Jackson Laboratory (RRID: IMSR_JAX:026622), and “NBSGW-SGM3” produced at the Francis Crick Institute by breeding for 7 generations NBSGW and NSG-SGM3 (NOD. Prkdc^scid^ Il2rg^tm1^ Tg (CMV-IL3, CSF2, KITLG). Mice were bred in isolators with aseptic standard operating procedures in the Biological Research Facility of The Francis Crick Institute. Once weaned, mice were kept in ventilated cages. All animal experiments were performed under the U.K Home Office project license (70/8904) and approved by The Francis Crick Institute animal ethics committee review board in accordance with the institute guidance and following the ARRIVE guideline. Male and female mice aged between 8-12 weeks, were used in these experiments. These mice were injected with human HSPCs (10,000-20,000 Lin-CD34+CD38- cells/mouse) via intravenous administration. Engraftment and mutation efficiency within the human hematopoietic system reconstituted were analyzed for each mouse by bone marrow aspiration at 8-10 weeks post injection, and the mice were sacrificed between 12-14 weeks post-transplantation or otherwise indicated.

### Strategies to analyze human TET2-derived clonal hematopoiesis (CH)

TET2^WT^ and TET2^Mut^ HSPCs were seeded in plates or injected into mice in competition or alone. We follow three different strategies. One, non-competitive setting, where TET2^WT^ or TET2^Mut^ HSPCs were seeded in separate wells to avoid confounding effects. Second, a competitive setting based on mixing and seeding together TET2^WT^ and TET2^Mut^ HSPC based on their different HLA type. Of note, we pooled at least two donors from each HLA type when performing these experiments and we have also switched the HLA types assigned to the TET2^WT^ or TET2^Mut^ HSPCs to rule out the influence of the different HLA type in our analysis. The third strategy consisted in a competitive setting but using the same pool of cord blood donors to generate the TET2^WT^ and TET2^Mut^ HSPCs. Human HSPCs were separated for CRISPR editing and after 48 hours and before the seeding were pooled together at a prefix ratio. In this case, TET2-CH frequency was measured by MiSeq at the specific time points indicated in the figures.

### Flow cytometry analysis and cell sorting of human HSPCs and immune progeny

All experiments were analyzed at the Flow Cytometry core facility of The Francis Crick Institute using the LSR FORTESSA (BD Biosciences) equipped with a 488-nm laser, a 561-nm laser, a 633-nm laser, and a 405-nm laser. For sorting, cell suspensions were filtered through a 35-µm nylon mesh (Falcon, Cat# 352235) and sorted in a BD FACS FUSION cell sorter (RRID:SCR_025715) equipped with 488-nm, 561-nm, 633-nm, and 405-nm lasers. The antibodies used to sort, and phenotype human HSPCs and immune progeny are listed in Supplementary Table 7. Exclusion of dead cells was done by staining with the fluorescent dye DAPI (1 µg/ml; BD Biosciences, Cat# 564907) and exclusion of remaining mouse hematopoietic cells by including anti-mouse CD45-BV410 (clone 30F11, BD Bioscience, RRID: AB_10899570) and gating out the positive cells. All experiments were analyzed with FACSDiva 6.2 (BD Biosciences, RRID:SCR_001456).

### RNA extraction, library preparation and RNA-sequencing of human HSPCs and monocytes

Sorted cell populations (CD34+ cells and CD14+ cells, see Figure 2) were pelleted and RNA was extracted using RNeasy Micro Kit (Qiagen, Cat#74004). Total RNA quality was verified in an Agilent 2100 Bioanalyzer (Agilent) and samples with RNA integrity number (RIN) of 8 or above were used prior to library preparation. Libraries were prepared using KAPA Stranded with RiboErase RNA-seq kit (Roche, Cat# 07962304001). Briefly, 17–25 ng of starting RNA was subjected first to cytoplasmic and mitochondrial ribosomal RNA (rRNA) depletion by hybridization of complementary DNA oligonucleotides, followed by treatment with RNase H and DNase to remove rRNA duplexed to DNA and original DNA oligonucleotides. Samples depleted of rRNA were then subjected to 94 °C for 6 min in the 2×Fragment, Prime, and Elute Buffer and we obtained 200–300 bp fragments. cDNA synthesis was run in two steps. The ligation step consisted of a final volume of 110 μl of the adaptor ligation reaction mixture with 60 μl of input cDNA, 5μl of diluted adaptor, and 45 μl of ligation mix (50 μl of ligation buffer + 10 μl of DNA ligase). The Kapa Dual- Indexed Adaptors stock concentration was diluted to 1.5mM to get the best adaptor concentration for library construction. The ligation cycle was run according to the manufacturer’s instructions. To remove short fragments such as adaptor dimers, 2X AMPure XP bead clean-ups were done (0.63 SPRI and 0.7 SPRI). To amplify the library, 15 PCR cycles were applied to the cDNA KAPA mix. Amplified libraries were purified using AMPure XP. The quality and fragment size distributions of the purified libraries were assessed with D1000 ScreenTape assay and reagents using Tapestation 42000 systems (Agilent Technologies, USA). Sequencing was then performed in a NovaSeq 6000 Sequencing System (Illumina, RRID:SCR_016387) with 25 million single-end 100 bp reads/sample. Obtained FASTQ files were uploaded to the R environment where adapter and quality trimming was performed, and a quality control report was generated. Alignment was performed using the Salmon strategy under the R environment where FASTQ files were aligned to the grch38 human genome under the Salmon reference format. Obtained raw counts were then uploaded and analyzed using the package Deseq2 on R. Data normalization, regularization, and batch correction was performed from which PCA charts and pairwise comparisons between control and mutated cells were obtained. RNA-Seq analysis of the different stages of the neutrophil development was carried out in R using DESEq2 using a model that accounted for replicate, litter, and experimental group. Gene counts were normalized using variance stabilized transformation (VST), which were used for PCA analysis using the plotPCA function of DESeq2. Differential genes between groups were determined within DESeq2 using the IHW method for independent hypothesis weighting and the ashr method for log fold change shrinkage. To produce heatmaps of top differentially expressed genes, for each comparison, genes with an adjusted p-value of <= 0.05 were selected and ranked by their log2 fold change values. 500 differential genes were selected from the top and bottom of these lists to capture equal numbers of up and down regulated genes. These gene lists were concatenated, the VST normalized counts retrieved for all samples and transformed to z-scores. These z-score values were plotted using the pheatmap package within R. Gene set enrichment analysis (GSEA), was performed within R using the fgsea package against the Hallmark pathways from the Molecular Signatures database, using the ranked stat values from the non-shrunken differential analysis results. Scatterplots comparing the log2 fold changes of selected cell-type comparisons against genotype comparisons were produced by collecting genes with adjusted p-values of <= 0.05 in the cell-type comparison and matching these genes with the results from the genotype comparison. Log2 fold changes for each gene for both comparisons could then be plotted as x vs y coordinates. A line of best fit was calculated using a linear model within R and plotted using ggplot2.

### In vivo models of stress for the analysis of human TET2-CH

To assess the impact of systemic inflammation, NBSGW humanized mice reconstituted with human HSPCs were injected intraperitoneally every other week with 1 μg/g of LPS derived from Escherichia coli (L4391, Sigma) or PBS from 6 weeks post human HSPCs injection for total of 10 injections. Mice were culled 24 hours after the last injection.

To assess the HSPC cell-intrinsic impact of LPS, we first challenge with LPS the mix of TET2^WT^ and TET2^Mut^ HSPCs in vitro (as done previously with 1μg/ml) and after 48 hours the mix of cells was injected in NBSGW mice.

To assess the influence of the irradiation as preconditioning of the bone marrow niche, we irradiated the NBSGW mice at 1.5 Gy, 24 hours before the injection of the mix of TET2^WT^ and TET2^Mut^ HSPCs.

### Analysis of TET2-CH in peripheral tissues and humanized ectopic scaffold

After 12-14 weeks of injection of human HSPCs, humanized NBSGW or NBSGW-SGM3 mice were culled and bones, blood, lung and spleen were harvested. Bone marrow was isolated by spinning of the long bones (tibia, femur and hip) for 30 s at 10,000 r.p.m. or by crashing all the main bones (long bones and spine, sternum and humerus) with a mortar before collecting a cell suspension with a micropipette and filtering using a 100μm cell strainer. 100 μl of peripheral blood were collected in heparin tubes and transfer to FACS tubes for red blood cel lysis. Lungs were cut into small pieces and digested with collagenase VIII (1 mg ml^−1^, Sigma-Aldrich) and DNase I (0.4 mg ml^−1^, Roche) in Roswell Park Memorial Institute (RPMI) 1640 medium for 20 min at 37°C. Spleens were cut into small pieces and smashed through a 100μm cell strainer (BD Biosciences). After centrifugation, digested tissues were resuspended in Red Blood cell lysis buffer for 10 min at 4°C and washed with FACS buffer (3% FCS and 5 mM EDTA in PBS).

Following our previous protocol (38), we also analyzed TET2-CH in a humanized ectopic model containing human mesenchymal stromal cells (MSCs) as support for human HSPCs. In brief, gelfoam collagen-based sponges (Pfizer, US) were cut into pieces of similar size (6.6 mm x 7.5 mm x 7 mm) with a sterile scalpel. Subsequently, previously expanded human MSCs where injected into scaffolds (50-100.000 cells in 50 µL/scaffold). Scaffolds were incubated in a plate at 37 °C in 5% CO_2_-humidified incubator, after 2 hours MSC media was gently added in each well containing hanging scaffolds and incubated overnight. The following day, we followed two strategies. (1) Scaffolds were implanted in mice to analyze TET2-CH recruitment into the scaffolds from intravenously injected human HSPCs or (2) scaffolds were injected with 7.000-12.000 human HSPCs in 50 µL/scaffold in Stem span SFEM II (Stem Cell Technologies, Canada) with 1% of penicillin/streptomycin (Sigma-Aldrich), containing 100 ng/mL SCF, 100 ng/mL FLT3-L and 100 ng/mL TPO (Peprotech). These scaffolds were then incubated 24-48 hours before subcutaneous implantation of the scaffold in mice to analyze the growth dynamic of TET2-CH in a human stromal environment. At the end of the experiment, scaffolds were retrieved and digested in Dispase, DNAse I (VWR) at 10µg/mL, Collagenase type I (Merck,) at 1mg/mL and 10% FBS (Sigma-Aldrich) solution. Digested scaffolds were then filtered through a sterile 5 mL tube with a cell strainer cap.

### Single cell RNA-sequencing (scRNA-seq) of human HSPC and immune progeny

TET2-CH humanized mice were subjected to a periodic regimen of LPS injections (see details above), at the end of the treatment, and 24 weeks after the injection of human HSPCs, mice were sacrificed, and bone marrow cells were retrieved by centrifugation, and we performed FACS of the human HSPC population (CD45^+^Lin^-^CD34^+^CD38^-^ CD45RA^-^). Human HSPCs were further separated based on the HLA type that allows us to separate and run the 10x independently for TET2^WT^ and TET2^Mut^ human at the Advanced sequencing facility of the Francis Crick Institute. HSPCs samples were prepared using 10X 3’ V3.1 and sequenced using the HiSeq 4000 (Illumina, RRID:SCR_016386), for immune progeny samples we used the 10X multiome kit and were sequenced in NovaSeq 6000 (Illumina, RRID:SCR_016387). The concentration and viability of the single cell suspension was measured using trypan blue using Eve Automatic Cell Counter. Approximately 3,000-10,000 cells were loaded on Chromium Chip and partitioned in nanoliter scale droplets using the Chromium Controller and Chromium Next GEM Single Cell Reagents (Chromium Single Cell 3' Reagent Kits User Guide (v3.1)). Within each droplet the cells were lysed, and the RNA was reverse transcribed. All the resulting cDNA within a droplet shared the same cell barcode. Illumina compatible libraries were generated from the cDNA using Chromium Next GEM Single Cell library reagents in accordance with the manufacturer’s instructions (10x Genomics, CG000315 Chromium Single Cell 3' Reagent Kits User Guide (v3.1 - Dual Index)). For QC of libraries, we used the Agilent TapeStation and sequenced using the Illumina HiSeq4000. Sequencing read configuration: 28-10-10-90. For the immune progeny, we sorted human hematopoietic cell subsets from humanized mice reconstituted with either TET2^WT^ or TET2^Mut^ HSPCs as described in Figure S5A. The concentration and viability of the single nuclei suspension was measured using acridine orange (AO) and propidium iodide (PI) and the Luna-FX7 Automatic Cell Counter. Approximately 15,000-20,000 nuclei were transposed, then loaded on Chromium Chip and partitioned in nanoliter scale droplets using the Chromium Controller and Chromium Next GEM Single Cell Reagents (Chromium Next GEM Single Cell Multiome ATAC + Gene Expression Reagent Kits User Guide, User Guide, CG000338). Sequencing read configuration: 28-10-10-90 (GEX). The 10x Barcodes in each library type are used to associate individual reads back to the individual partitions, and thereby, to each individual nucleus.

### scRNA-seq raw data processing

For the HSPC single cell RNA-seq data, FASTQ files from different lanes were merged and STAR v. 2.7.10b(60) (RRID:SCR_004463) was used to align each of them to the Ensembl h38 genome (release 108) using the following parameters: --runMode alignReads, --soloType CB_UMI_Simple, --soloUMIlen 12, --runRNGseed 1. For the myeloid progeny single cell RNA-seq data, cellranger v. 7.1.0 (RRID:SCR_023897) was used using pre-computed references (“refdata-cellranger-GRCh38-3.0.0”) and chemistry=ARC-v1. Filtered reads were then imported into R 4.3.2 as a SingleCellExperiment object using the TENxIO R package v. 1.4.0. Data from HSPCs and myeloid progeny were processed separately and never integrated together. QC statistics were calculated for each sample separately, using the isOutlier function from the scran R/BioConductor package (61) v 1.30.2 to flag cells to be discarded, using the following cut-off points: 3 Median Absolute Deviations (MADs) for log10(total UMI counts) and total genes detected above and below and 3 MADs for % mitochondrial, ribosomal and MALAT1 transcripts, above. The final number of cells passing QC filtering was 4136 cells (HSPCs) and 28026 cells (myeloid progeny). Counts matrices were normalized using batch-aware normalization through the multiBatchNorm() function in the batchelor R/BioConductor package(62) v. 1.18.1.

### Data integration, clustering and differential expression and pathway analysis

Highly variable genes were identified using batch-aware mean-variance modelling through the modelGeneVar function from scran, which fits a mean-variance trend to each sample individually and then combines variances and p-values for prioritization. The top 2000 most highly variable genes were used in all cases. These were used to subset the log-normalized counts as input for Principal Component Analysis (PCA)-mediated dimensionality reduction, obtained through the runPCA() function from the scater R/BioConductor package (63) v 1.30.1 (RRID:SCR_001905). The first 20 components were used as an input for UMAP embedding (64) implemented through the uwot R package v. 0.1.16, using the following parameters: min_dist = 0.7, n_neighbors = 64. HSPC single cell transcriptomes were integrated across samples using the fastMNN() function from batchelor, with default parameters, using the first 20 dimensions of the PCA space as input. In the resulting corrected space, 20 dimensions were used as well for UMAP embedding as described above. A shared-nearest neighbor graph (SNN) was built in the corrected space using 10 neighbors for Jaccard weightage of the edges, through the makeSNNGraph() function from the bluster R/BioConductor package v. 1.12.0. Multi-resolution clustering using 5 equally spaced values of resolution (from 0.01 to 0.1) was run on the SNN graph using Leiden clustering as implemented in the cluster_leiden() function from the igraph R package v. 2.0.1.1 with 40 iterations, and clusters were linked together into metaclusters using the linkClusters() function from bluster. Gene regulatory network analysis was performed on the HSPC dataset using pySCENIC v0.9.18 (65,66) using default parameters and lists of transcription factors, ranking databases and motif databases downloaded from the SCENIC website to identify 189 regulons, co-expression modules of transcription factors and target genes. All genes differentially expressed (Adjusted p value <0.05, Log2FC > 0.5, mean normalized expression >0.2) in the HSPC cluster between LPS-treated WT condition and LPS-treated TET2 condition were subjected to gene set enrichment analysis using gseapy against the transcription factor target gene lists. Cellular enrichment of regulon activity (AUCell) was calculated in pySCENIC and differential expression of regulon activity was calculated in scanpy using a two tailed t-test with Benjamini-Hochberg correction. To validate differential transcription factor regulon activity, transcription factor target genes were downloaded from the EnrichR website from the gene sets ‘eGFP-JUNB K562 hg19 from ENCODE_TF_ChIP-seq_2015’ and ‘FOS K562 hg19 from ENCODE_TF_ChIP-seq_2015’; gene set enrichment scores were computed per cell in scanpy as the average expression of the gene set after subtraction of the expression of a reference set of genes selected at random from the gene pool for each binned expression value. Differential activity of gene set enrichment scores between conditions in HSPC cluster was calculated using a two-tailed t-test.

For myeloid progeny analysis, single cell transcriptomes were integrated using fastMNN() as for the HSPC dataset. The clustering strategy was also similar, except the SNN graph was built using 20 neighbors and clustered using resolutions of 0.2, 0.4, 0.5, 0.6, 0.8, and 1. Clusters were not linked but rather evaluated using a clustering tree via the clustree R package (67) v. 0.5.1, and a final resolution of 0.5 was provisionally chosen as a good compromise between over- and under-clustering. Cluster-level DE was performed in both the HSPC and the Myeloid progeny dataset using a Wilcoxon rank-sum test on log-normalized counts, as implemented in the wilcoxauc() function from the presto R package v 1.0.0. The same function was used for marker finding, and genes were ranked by difference between their expression in one cluster versus all the other ones. Gene Set Enrichment Analysis (GSEA) was run against pathways in the Hallmark and Reactome collections from the Molecular Signature DataBase (MsigDB) through the fgsea R package v.1.28.0. For the HSPC results, the heatmap of averaged log-normalized counts was drawn using the ComplexHeatmap R package (68) v. 2.18.0. The myeloid dataset was projected onto the MoMac-VERSE dataset (45) using Run.ProjecTILs() from the ProjecTILs R package(69) v. 3.3.0 with default parameters. The MoMac-VERSE dataset was randomly sub-sampled taking 10% of the original cell number within each cluster identified by the authors. Moreover, since the UMAP model was not available for the original MoMac-VERSE dataset, it was recalculated with uwot to embed myeloid precursors as well.

### Epigenome analysis of human TET2^Mut^ HSPCs

For methylome analysis, we generated CRISPR-edited Lineage^-^CD34^+^CD38^-^ cells from 3 independent pools of cord blood donors. Four days after the introduction of the mutation, we harvested TET2^WT^ or TET2^Mut^ cells and perform DNA extraction. We run the MethylationEPIC array (Illumina) using 500 ng of DNA. HSPCIDAT files from Infinium MethylationEPIC v2 array were processed using the SeSAMe package (v1.24.0) in R, to convert raw signal data to beta values. Briefly, for each sample, the probe detection P values were calculated using the pOOBAH algorithm and poor-quality probes were masked. Data was normalized using noob to remove background signal and dyeBiasNL for dye bias correction. Finally, signal intensities were summarized into beta values using the getBetas function. Of 937690 probes in the array, 863337 were retained across all 12 samples. Differentially methylated probes were analyzed using limma (v3.62.0). Volcano plots were produced using -log10(p-value) and methylation change for pairwise comparisons. Hypermethylated probes in Tet2Mut for both CTRL and LPS conditions were annotated for associated genes or transcription factor binding sites using Illumina Human Methylation EPICv2anno.20a1.hg38 (v1.0.0). Transcription factor binding site enrichment was calculated using testEnrichment as part of the SeSAMe package. Estimate refers to log2(odds ratio). Overlap corresponds to number of probes in our list of hypermethylated probes that are present in the list of all probes in the array associated with each transcription factor binding site. GSEA enrichment analysis (GSEA) was performed using fgsea in R (v1.32.0) against the Hallmark pathways from the Molecular Signatures database. Genes associated with hypermethylated probes (methylation change >10%, adjusted p-value < 0.05) were ranked based on magnitude of methylation change. For genes of interest, associated probes were identified using sesameData_getProbesByGene. Probes were mapped to specific regions of the gene using IlluminaHumanMethylationEPICv2manifest. Mean beta values for each treatment group were used to produce a heatmap using pheatmap (v1.0.12) or line graph. For all analysis, data was visualized using SeSAMe package, ggplot2 (v3.5.1) and Inkscape.

For ATACSeq analysis we sorted Lineage^-^CD34^+^CD38^-^CD45RA^-^ cells from bone marrow of humanized mice engrafted with a mix of TET2^WT^ and TET2^Mut^ HSPCs. We isolated and performed DNA extraction from 25,000 TET2^WT^ cells (n =4) and TET2^Mut^ cells (n= 7). Improved OMINI-ATAC protocol was followed as described previously (70). Library PCR amplification was done with 9 cycles and double-sided size selection was performed using KAPA Pure beads (Roche, 07983280001). Sequencing was then performed in a HiSeq4000 Sequencing System (Illumina) with a loading molarity of 200pM plus 10% Phix (illumine). Each sample was sequenced to achieve 30 million Pair-end 100 bp reads/sample. For the analysis of percentage of fragmented DNA and differentially annotated peaks we use the nf-core pipeline within the nextflow bioinformatics workflow framework (version 19.10.0). FASTQ files were aligned to the GRCh38 human genome. Peaks were called using MACS2 with the –narrow_peak parameter. Peaks were annotated using HOMER and a consensus set of peaks, merged between replicates from the same condition, was generated using BEDTools. Differential accessibility analysis between conditions was performed using DESeq2; peaks with a false discovery rate of 0.05 were considered differentially accessible. Transcription factor motif enrichment was performed on differentially accessible peaks using the Motif analysis module from the RGT suite (71). Motifs with a corrected p value of <0.0001 were considered significantly enriched.

### Quantification and statistical analysis

Statistical analyses were performed using GraphPad Prism software (RRID:SCR_002798). Results are depicted with the individual values and indicating mean ± SEM in each case. The statistical test used is specified in each figure legend.

### Materials availability

All biological materials used in this study are available from the lead contact upon request or from commercial sources. This study did not generate new unique reagents.

### Lead contact

Further information and requests for resources and reagents should be directed to and will be fulfilled by the lead contact, Prof. Dominique Bonnet, email: (Dominique.bonnet@crick.ac.uk).

## Supplementary Material

1

2

3

4

5

6

7

8

## Data Availability

Bulk RNA sequencing and single cell RNA sequencing data are deposited in the NCBI Gene Expression Omnibus (GEO) database under accession number GSE287524 for bulk RNASeq and GSE285379 for scRNASeq and GSE307624 for ATAC-Seq. All other data that support the findings of this study are available in the article Supplementary Information files or from the corresponding author upon request.

## Statement of significance

This study reveals that stress conditions inducing emergency myelopoiesis expand human TET2-mutant clones. TET2 mutation trigger an epigenetic silencing of AP-1 factors contributing to dampen the inflammatory responsiveness in TET2^mut^ HSPCs while, simultaneously promoting hyperinflammatory TET2^mut^ monocyte–macrophage activation and differentiation, reinforcing inflammation.

## References

[1] Fabre MA, de Almeida JG, Fiorillo E, Mitchell E, Damaskou A, Rak J (2022). The longitudinal dynamics and natural history of clonal haematopoiesis. Nature.

[2] Mitchell E, Spencer Chapman M, Williams N, Dawson KJ, Mende N, Calderbank EF (2022). Clonal dynamics of haematopoiesis across the human lifespan. Nature.

[3] Jaiswal S, Ebert BL (2019). Clonal hematopoiesis in human aging and disease. Science.

[4] Karpova D, Huerga Encabo H, Donato E, Calderazzo S, Scherer M, Llorian-Sopena M (2025). Clonal hematopoiesis landscape in frequent blood donors. Blood.

[5] Pardali E, Dimmeler S, Zeiher AM, Rieger MA (2019). Clonal hematopoiesis, aging, and cardiovascular diseases. Exp Hematol.

[6] Bowman RL, Busque L, Levine RL (2018). Clonal Hematopoiesis and Evolution to Hematopoietic Malignancies. Cell Stem Cell.

[7] Jaiswal S, Fontanillas P, Flannick J, Manning A, Grauman PV, Mar BG (2014). Age-Related Clonal Hematopoiesis Associated with Adverse Outcomes. N Engl J Med.

[8] Bhattacharya R, Zekavat SM, Haessler J, Fornage M, Raffield L, Uddin MM (2021). Clonal Hematopoiesis Is Associated with Higher Risk of Stroke. Stroke.

[9] Kar SP, Quiros PM, Gu M, Jiang T, Mitchell J, Langdon R (2022). Genome-wide analyses of 200,453 individuals yield new insights into the causes and consequences of clonal hematopoiesis. Nat Genet.

[10] Miller PG, Qiao D, Rojas-Quintero J, Honigberg MC, Sperling AS, Gibson CJ (2022). Association of clonal hematopoiesis with chronic obstructive pulmonary disease. Blood.

[11] Fuster JJ, MacLauchlan S, Zuriaga MA, Polackal MN, Ostriker AC, Chakraborty R (2017). Clonal hematopoiesis associated withTET2 deficiency acceleratesatherosclerosis development in mice. Science.

[12] Köhnke T, Majeti R (2021). Clonal Hematopoiesis: From Mechanisms to Clinical Intervention. Cancer Discov.

[13] Florez MA, Tran BT, Wathan TK, DeGregori J, Pietras EM, King KY (2022). Clonal hematopoiesis: Mutation-specific adaptation to environmental change. Cell Stem Cell.

[14] Caiado F, Pietras EM, Manz MG (2021). Inflammation as a regulator of hematopoietic stem cell function in disease, aging, and clonal selection. J Exp Med.

[15] Moran-Crusio K, Reavie L, Shih A, Abdel-Wahab O, Ndiaye-Lobry D, Lobry C (2011). Tet2 Loss Leads to Increased Hematopoietic Stem Cell Self-Renewal and Myeloid Transformation. Cancer Cell.

[16] Ito K, Lee J, Chrysanthou S, Zhao Y, Josephs K, Sato H (2019). Non-catalytic Roles of Tet2 Are Essential to Regulate Hematopoietic Stem and Progenitor Cell Homeostasis. Cell Rep.

[17] Meisel M, Hinterleitner R, Pacis A, Chen L, Earley ZM, Mayassi T (2018). Microbial signals drive pre-leukaemic myeloproliferation in a Tet2-deficient host. Nature.

[18] Caiado F, Kovtonyuk LV, Gonullu NG, Fullin J, Boettcher S, Manz MG (2023). Aging drives Tet2+/− clonal hematopoiesis via IL-1 signaling. Blood.

[19] Nakauchi Y, Azizi A, Thomas D, Corces MR, Reinisch A, Sharma R (2022). The Cell Type–Specific 5hmC Landscape and Dynamics of Healthy Human Hematopoiesis and TET2-Mutant Preleukemia. Blood Cancer Discov.

[20] Köhnke T, Feng Y, Majeti R (2024). A new era of functional experimentation in human hematopoiesis and leukemia research. Exp Hematol.

[21] Trowbridge JJ, Starczynowski DT (2021). Innate immune pathways and inflammation in hematopoietic aging, clonal hematopoiesis, and MDS. J Exp Med.

[22] Huerga Encabo H, Aramburu IV, Garcia-Albornoz M, Piganeau M, Wood H, Song A (2023). Loss of TET2 in human hematopoietic stem cells alters the development and function of neutrophils. Cell Stem Cell.

[23] Demel UM, Lutz R, Sujer S, Demerdash Y, Sood S, Grunschlager F (2022). A complex proinflammatory cascade mediates the activation of HSCs upon LPS exposure in vivo. Blood Adv.

[24] Liu J, Guo YM, Hirokawa M, Iwamoto K, Ubukawa K, Michishita Y (2012). A synthetic double-stranded RNA, poly I: C, induces a rapid apoptosis of human CD34+ cells. Exp Hematol.

[25] Wilkinson AC, Ishida R, Kikuchi M, Sudo K, Morita M, Crisostomo RV (2019). Long-term ex vivo haematopoietic-stem-cell expansion allows nonconditioned transplantation. Nature.

[26] Wilkinson AC, Ishida R, Nakauchi H, Yamazaki S (2020). Long-term ex vivo expansion of mouse hematopoietic stem cells. Nat Protoc.

[27] Cull AH, Snetsinger B, Buckstein R, Wells RA, Rauh MJ (2017). Tet2 restrains inflammatory gene expression in macrophages. Exp Hematol.

[28] Li H, Guan Y, Liang B, Ding P, Hou X, Wei W (2022). Therapeutic potential of MCC950, a specific inhibitor of NLRP3 inflammasome. Eur J Pharmacol.

[29] Coll RC, Hill JR, Day CJ, Zamoshnikova A, Boucher D, Massey NL (2019). MCC950 directly targets the NLRP3 ATP-hydrolysis motif for inflammasome inhibition. Nat Chem Biol.

[30] Johnson CS, Williams M, Sham K, Belluschi S, Ma W, Wang X (2024). Adaptation to ex vivo culture reduces human hematopoietic stem cell activity independently of the cell cycle. Blood.

[31] Williams MJ, Wang X, Bastos HP, Grondys-Kotarba G, Wu Q, Jin S (2025). Maintenance of hematopoietic stem cells by tyrosine-unphosphorylated STAT5 and JAK inhibition. Blood Adv.

[32] Sano S, Oshima K, Wang Y, MacLauchlan S, Katanasaka Y, Sano M (2018). Tet2-Mediated Clonal Hematopoiesis Accelerates Heart Failure Through a Mechanism Involving the IL-1β/NLRP3 Inflammasome. J Am Coll Cardiol.

[33] Buscarlet M, Provost S, Zada YF, Bourgoin V, Mollica L, Dubé MP (2018). Lineage restriction analyses in CHIP indicate myeloid bias for TET2 and multipotent stem cell origin for DNMT3A. Blood.

[34] Ko M, Bandukwala HS, An J, Lamperti ED, Thompson EC, Hastie R (2011). Ten-eleven-translocation 2 (TET2) negatively regulates homeostasis and differentiation of hematopoietic stem cells in mice. Proc Natl Acad Sci U S A.

[35] Cimmino L, Dolgalev I, Wang Y, Yoshimi A, Martin GH, Wang J (2017). Restoration of TET2 Function Blocks Aberrant Self-Renewal and Leukemia Progression. Cell.

[36] Barbieri D, Elvira-Matelot E, Pelinski Y, Genève L, de Laval B, Yogarajah G (2018). Thrombopoietin protects hematopoietic stem cells from retrotransposon-mediated damage by promoting an antiviral response. J Exp Med.

[37] Cao X, Wu X, Frassica D, Yu B, Pang L, Xian L (2011). Irradiation induces bone injury by damaging bone marrow microenvironment for stem cells. Proc Natl Acad Sci U S A.

[38] Abarrategi A, Foster K, Hamilton A, Mian SA, Passaro D, Gribben J (2017). Versatile humanized niche model enables study of normal and malignant human hematopoiesis. J Clin Invest.

[39] Nagata Y, Nishida E, Todokoro K (1997). Activation of JNK signaling pathway by erythropoietin, thrombopoietin, and interleukin-3. Blood.

[40] Matsuguchi T, Masuda A, Sugimoto K, Nagai Y, Yoshikai Y (2003). JNK-interacting protein 3 associates with Toll-like receptor 4 and is involved in LPS-mediated JNK activation. EMBO J.

[41] Arndt PG, Suzuki N, Avdi NJ, Malcolm KC, Worthen GS (2004). Lipopolysaccharide-induced c-Jun NH2-terminal kinase activation in human neutrophils: Role of phosphatidylinositol 3-kinase and Syk-mediated pathways. J Biol Chem.

[42] Adunyah SE, Unlap TM, Wagner F, Kraft AS (1991). Regulation of c-jun expression and AP-1 enhancer activity by granulocyte-macrophage colony-stimulating factor. J Biol Chem.

[43] Wang Y, Zhou C, Huo J, Ni Y, Zhang P, Lu C (2015). TRAF6 is required for the GM-CSF-induced JNK, p38 and Akt activation. Mol Immunol.

[44] Montagner S, Leoni C, Emming S, Della Chiara G, Balestrieri C, Barozzi I (2016). TET2 Regulates Mast Cell Differentiation and Proliferation through Catalytic and Non-catalytic Activities. Cell Rep.

[45] Mulder K, Patel AA, Kong WT, Piot C, Halitzki E, Dunsmore G (2021). Cross-tissue single-cell landscape of human monocytes and macrophages in health and disease. Immunity.

[46] Cao R, Thatavarty A, King KY (2024). Forged in the fire: Lasting impacts of inflammation on hematopoietic progenitors. Exp Hematol.

[47] Spencer Chapman M, Wilk CM, Boettcher S, Mitchell E, Dawson K, Williams N (2024). Clonal dynamics after allogeneic haematopoietic cell transplantation. Nature.

[48] Cai Z, Kotzin JJ, Ramdas B, Chen S, Nelanuthala S, Palam LR (2018). Inhibition of Inflammatory Signaling in Tet2 Mutant Preleukemic Cells Mitigates Stress-Induced Abnormalities and Clonal Hematopoiesis. Cell Stem Cell.

[49] Jakobsen NA, Turkalj S, Zeng AGX, Stoilova B, Metzner M, Rahmig S (2024). Selective advantage of mutant stem cells in human clonal hematopoiesis is associated with attenuated response to inflammation and aging. Cell Stem Cell.

[50] Zioni N, Bercovich AA, Chapal-Ilani N, Bacharach T, Rappoport N, Solomon A (2023). Inflammatory signals from fatty bone marrow support DNMT3A driven clonal hematopoiesis. Nat Commun.

[51] Reed SC, Croessmann S, Park BH (2023). CHIP Happens: Clonal hematopoiesis of indeterminate potential and its relationship to solid tumors. Clin Cancer Res.

[52] Coombs CC, Gillis NK, Tan X, Berg JS, Ball M, Balasis ME (2018). Identification of clonal hematopoiesis mutations in solid tumor patients undergoing unpaired next-generation sequencing assays. Clin Cancer Res.

[53] Pich O, Bernard E, Zagorulya M, Rowan A, Pospori C, Slama R (2025). Tumor-Infiltrating Clonal Hematopoiesis. N Engl J Med.

[54] Pan W, Zhu S, Qu K, Meeth K, Cheng J, He K (2017). The DNA Methylcytosine Dioxygenase Tet2 Sustains Immunosuppressive Function of Tumor-Infiltrating Myeloid Cells to Promote Melanoma Progression. Immunity.

[55] Hsiehchen D, Sfreddo HJ, Zhao K, Han CY, Morris LGT (2022). Clonal hematopoiesis and differential outcomes after immune checkpoint blockade. Cancer Cell.

[56] Nguyen YTM, Fujisawa M, Nguyen TB, Suehara Y, Sakamoto T, Matsuoka R (2021). Tet2 deficiency in immune cells exacerbates tumor progression by increasing angiogenesis in a lung cancer model. Cancer Sci.

[57] Huerga Encabo H, Ulferts R, Sharma A, Beale R, Bonnet D (2021). Infecting human hematopoietic stem and progenitor cells with SARS-CoV-2. STAR Protoc.

[58] Di Tullio A, Rouault-Pierre K, Abarrategi A, Mian S, Grey W, Gribben J (2017). The combination of CHK1 inhibitor with G-CSF overrides cytarabine resistance in human acute myeloid leukemia. Nat Commun.

[59] Meaker GA, Wilkinson AC (2024). Ex vivo hematopoietic stem cell expansion technologies: recent progress, applications, and open questions. Exp Hematol.

[60] Dobin A, Davis CA, Schlesinger F, Drenkow J, Zaleski C, Jha S (2012). STAR: ultrafast universal RNA-seq aligner. Bioinformatics.

[61] Lun ATL, McCarthy DJ, Marioni JC (2016). A step-by-step workflow for low-level analysis of single-cell RNA-seq data with Bioconductor. F1000Research.

[62] Haghverdi L, Lun ATL, Morgan MD, Marioni JC (2018). Batch effects in single-cell RNA-sequencing data are corrected by matching mutual nearest neighbors. Nat Biotechnol.

[63] McCarthy DJ, Campbell KR, Lun ATL, Wills QF, Hofacker I (2017). Scater: pre-processing, quality control, normalization and visualization of single-cell RNA-seq data in R. Bioinformatics.

[64] McInnes L, Healy J, Saul N, Großberger L (2018). UMAP: Uniform Manifold Approximation and Projection. J Open Source Softw.

[65] Aibar S, González-Blas CB, Moerman T, Huynh-Thu VA, Imrichova H, Hulselmans G (2017). SCENIC: Single-cell regulatory network inference and clustering. Nat Methods.

[66] Van de Sande B, Flerin C, Davie K, De Waegeneer M, Hulselmans G, Aibar S (2020). A scalable SCENIC workflow for single-cell gene regulatory network analysis. Nat Protoc.

[67] Zappia L, Oshlack A (2018). Clustering trees: a visualization for evaluating clusterings at multiple resolutions. Gigascience.

[68] Gu Z, Eils R, Schlesner M (2016). Complex heatmaps reveal patterns and correlations in multidimensional genomic data. Bioinformatics.

[69] Andreatta M, Corria-Osorio J, Müller S, Cubas R, Coukos G, Carmona SJ (2021). Interpretation of T cell states from single-cell transcriptomics data using reference atlases. Nat Commun.

[70] Corces MR, Trevino AE, Hamilton EG, Greenside PG, Sinnott-Armstrong NA, Vesuna S (2017). An improved ATAC-seq protocol reduces background and enables interrogation of frozen tissues. Nat Methods.

[71] Li Z, Kuo CC, Ticconi F, Shaigan M, Gehrmann J, Gusmao EG (2023). RGT: a toolbox for the integrative analysis of high throughput regulatory genomics data. BMC Bioinformatics.

