# Supplementary material for: Human TET2-mutant clonal hematopoiesis expansion is driven by distinct inflammatory signaling responses in stem cells versus myeloid progeny
Source: Blood Cancer Discov. Author manuscript; Available in PMC 2025 Dec 28. (PMC7618546; doi:10.1158/2643-3230.BCD-25-0070)
Supplement: 8 [file EMS211410-supplement-8.pdf]

Supplemental information

Supplementary Figures S1–S7 (see below).

Table S1. Table S1 provides the GSEA of Untreated compared to LPS conditions, related to Figure 2.

Table S2. Table S2 provides list of differentially expressed genes, related to Figure 2.

Table S3. Table S3 provides the GSEA of $TET2^{WT}$ compared to $TET2^{Mut}$ cells, related to Figure 2.

Table S4. Table S4 provides the complete list of transcription factors activated in human HSPCs from LPS-treated humanized mice.

Table S5. Table S5 provides the complete list of differentially methylated regions between $TET2^{WT}$ and $TET2^{Mut}$ HSPCs.

Table S6. Table S6 provides the complete list of differentially accessible transcription factor motifs from the ATAC-Seq analysis.

Table S7. Table S7 provides the list of antibodies used in flow cytometry and FACS.

Supplementary Figure S1

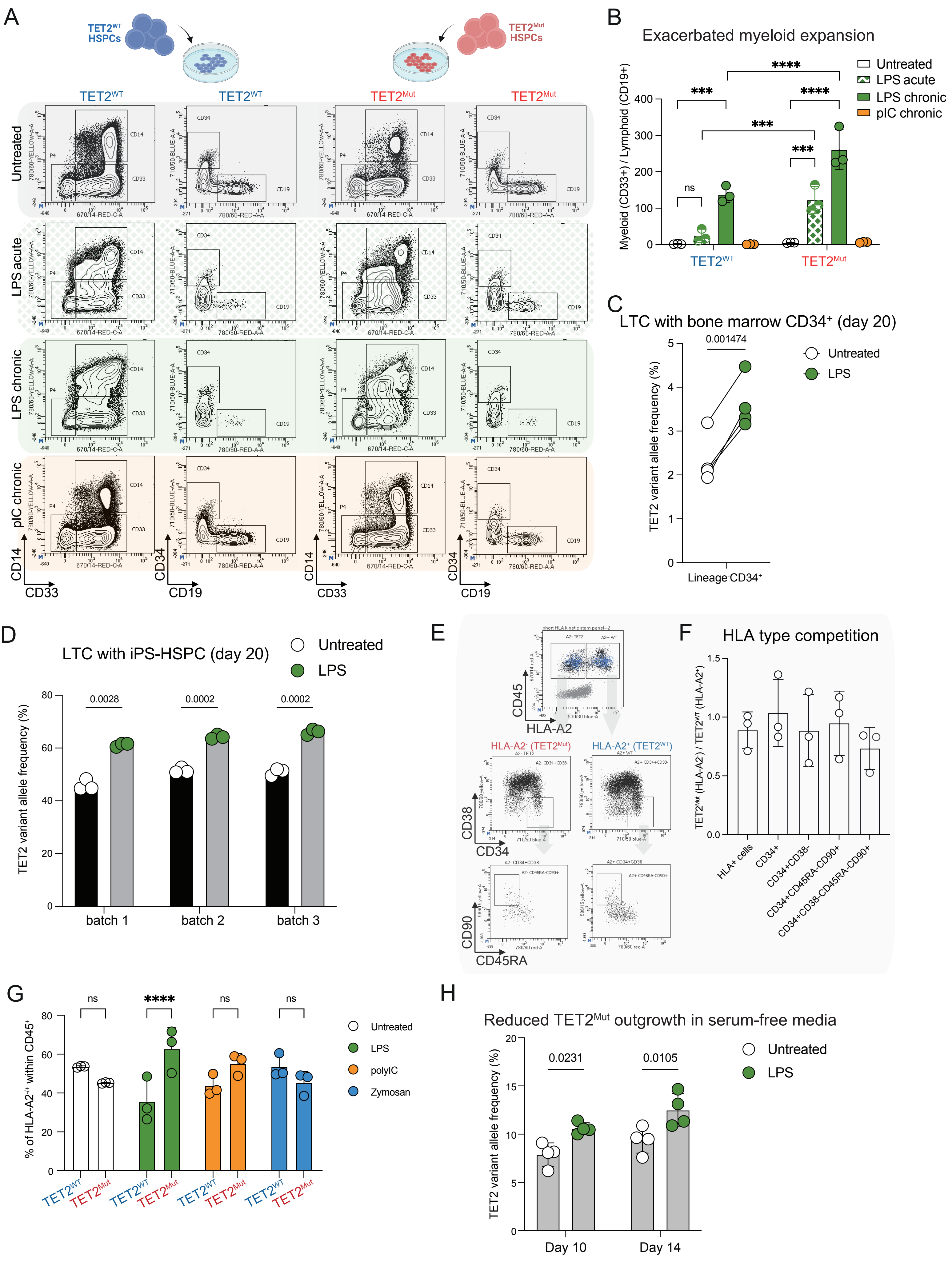

Figure S1. Long term culture competition assays of $TET2^{Mut}$ human HSPCs, related to Figure 1.

A. Representative flow cytometry gating strategy to analyze hematopoietic cell populations derived from $TET2^{WT}$ or $TET2^{Mut}$ HSCs upon different stress conditions.

B. Cell ratio between percentage of $CD33^+$ and $CD33^-CD19^+$ within $CD45^+$ cells in each $TET2^{WT}$ or $TET2^{Mut}$ derived LTC (see Figure S1C). Each dot represents a technical replicate for the same experiment performed with a pool of different biological donors. Three independent experiments using three different pools of biological donors were performed obtaining similar results. Data showing mean and SD from three technical replicates. Two-way ANOVA test used for significance, ns (non-significant); *** $p<0.005$; **** $p<0.001$.

C. Variant allele frequency (VAF) of TET2 mutations at day 20 of the LTC done from CRISPR-edited bone marrow CD34+ cells. Each paired of dots represents one biological bone marrow donor in untreated or after treatment with LPS. Paired t-test was used for significance and p-value is shown in the figure.

D. Variant allele frequency (VAF) of TET2 mutations at day 20 of the LTC done from HSPCs derived from CRISPR-edited human iPSCs. Monoclonal WT or $TET2^{Mut}$ iPSC-HSPCs were mixed at 1:1 ratio and bulk $CD45^+$ cell population was sequenced after 20 days of the competitive assay. We performed the experiment from 3 independent iPSC-HSPC clones (batch). Each dot represents a technical replicate. Unpaired t-test was used for significance and p-value are shown in the figure.

E. Representative flow cytometry gating strategy to analyze HLA-based competition assays between $TET2^{WT}$ and $TET2^{Mut}$ human HSPCs. We have validated and performed different LTC competition assays switching the HLA type assigned to $TET2^{Mut}$ HSPC to ensure no intrinsic bias associated to the difference in HLA type influence the outgrowth dynamic of $TET2^{Mut}$ HSPC.

F. Quantification of percentage of different HSPC compartments, Lin^-CD34^+; Lin^- $CD34^+CD38^-$; $Lin^-CD34^+CD45RA^-CD90^+$ including HSCs defined as $CD45^+$ $CD34^+CD38^-CD45RA^-CD90^+$ showing no significant difference between different HLA type donors. Each dot represents a technical replicate for the same experiment performed with a pool of different biological donors for each HLA type. Two-way ANOVA test used for significance.

G. Quantification of the percentage of different HLA types at the end of the competition assay. Each dot represents a technical replicate for the same experiment performed with a pool of different biological donors for each HLA type. Data showing mean and

SD from three technical replicates. Two-way ANOVA test used for significance, ns (non-significant); **** $p<0.001$.

H. Variant allele frequency (VAF) of TET2 mutations at day 10 and day 14 of the serum-free media condition assay. Each dot represents a technical replicate. Two independent experiments using different pools of biological donors were performed obtaining similar results. Two-way ANOVA test used for significance, p-values are shown in the figure.

Supplementary Figure S2

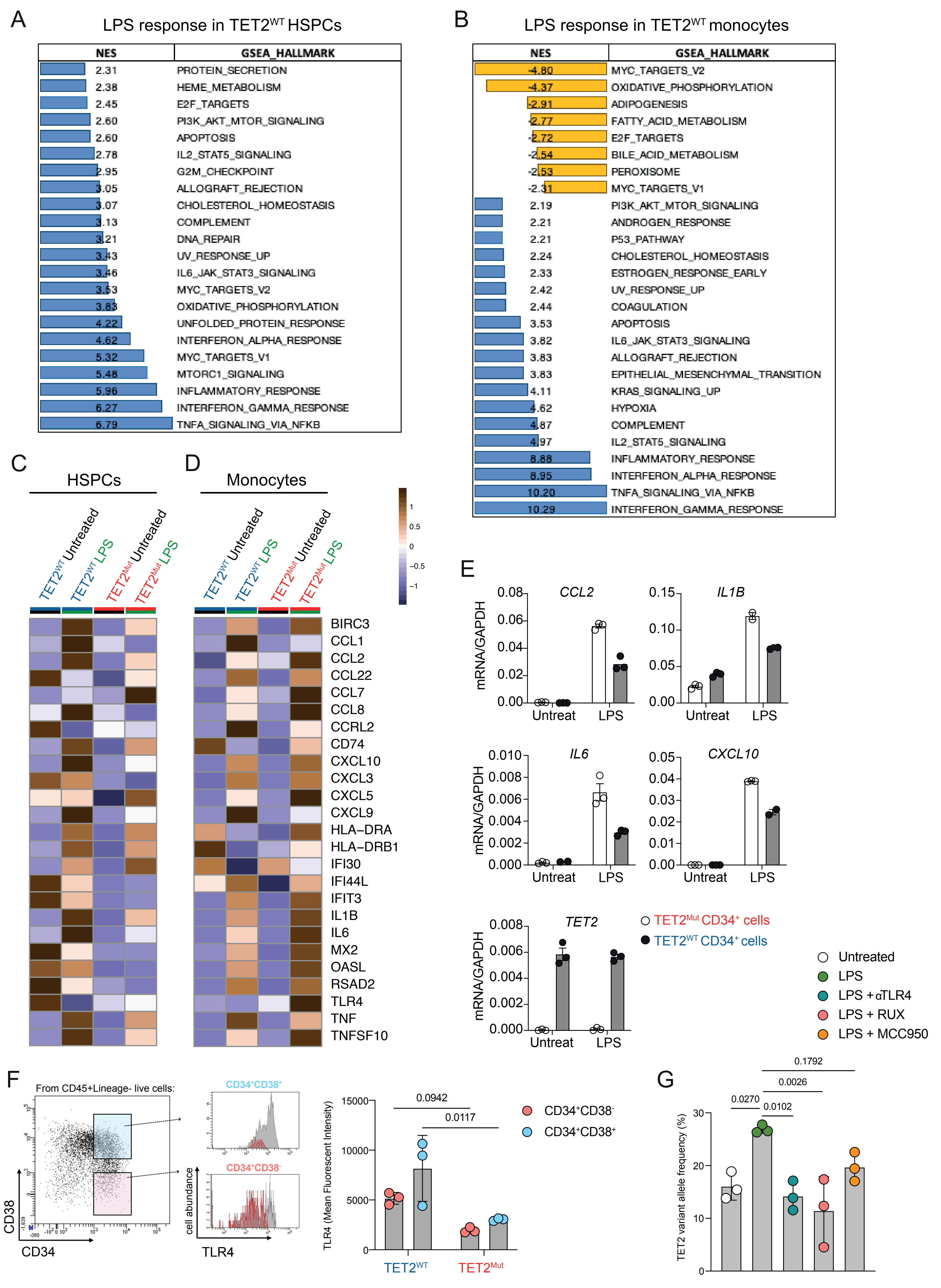

Figure S2. Transcriptional responses of human HSPCs and its myeloid progeny upon LPS challenge, related to Figure 2.

A. Gene Set Enrichment Analysis (GSEA) using Hallmark database for the comparison between untreated and LPS-treated $TET2^{WT}$ $CD34^{+}$ (see Supplementary Table 1).

B. GSEA using Hallmark database for the comparison between untreated and LPS-treated $TET2^{Mut}$ $CD34^{+}$ (see Supplementary Table 1).

C. GSEA using Hallmark database for the comparison between untreated and LPS-treated $TET2^{WT}$ $CD14^{+}$ (see Supplementary Table 1).

D. GSEA using Hallmark database for the comparison between untreated and LPS-treated $TET2^{Mut}$ $CD14^{+}$ (see Supplementary Table 1).

C-D. Heatmap of representative genes differentially expressed (adj. p.value < 0.05) after LPS stimulation (6 hours, 1ug/ml). (C) shows expression in $CD34^{+}$ cells and (D) shows expression in $CD14^{+}$ cells. See Supplementary Table 2 for complete list of differentially expressed genes.

E. Real-time quantitative PCR of different proinflammatory cytokines and TET2 in CD34+ cells from an independent experiment validating dampened upregulation of proinflammatory cytokines in $TET2^{Mut}$ HSPCs. Each dot represents a technical replicate for the same experiment performed with a pool of different biological donors for each HLA type. Data showing mean and SD from three technical replicates.

F. Quantification by flow cytometry of the TLR4 expression in $TET2^{WT}$ and $TET2^{Mut}$ $CD34^{+}CD38^{-}$ or $CD34^{+}CD38^{+}$ at day 14 of the LTC assay. Data showing mean fluorescent intensity (MFI) and SD from three technical replicates. Two-way ANOVA test used for significance, p-values are shown in the figure.

G. Variant allele frequency (VAF) of TET2 mutations at day 20 of the LTC done in the present of TLR4 blocking antibody, Ruxolitinib or MCC950. Data showing mean and SD from three technical replicates. Two-way ANOVA test used for significance, p-values are shown in the figure.

Supplementary Figure S3

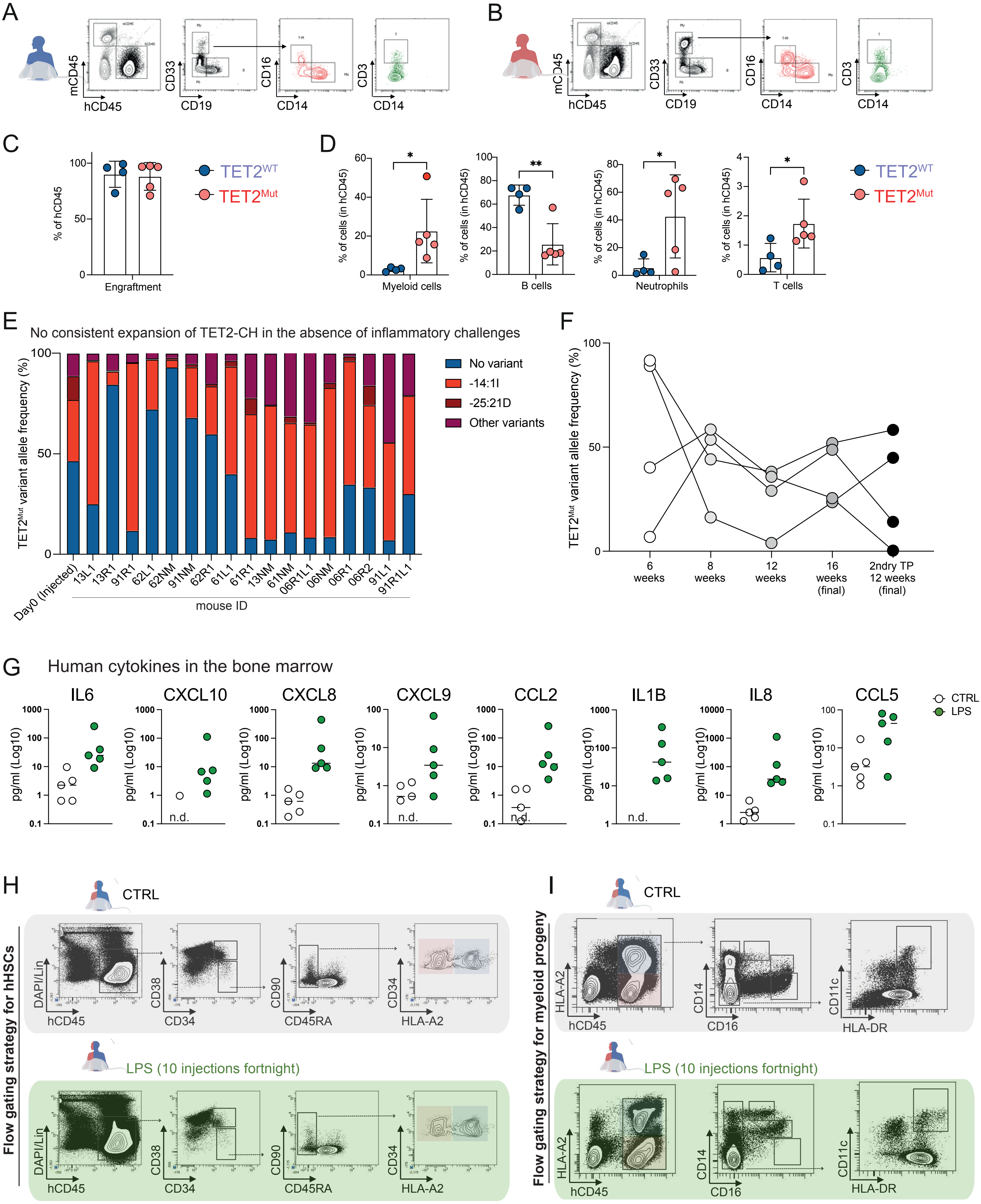

Figure S3. Absence of selective advantage of $TET2^{Mut}$ HSPCs in humanized NBSGW mice, related to Figure 3.

A-B. Representative flow cytometry gating strategy to analyze human hematopoietic reconstitution from $TET2^{WT}$ (A) or $TET2^{Mut}$ (B) human HSPCs in conventional NBSGW.

C. Quantification of human engraftment in the bone marrow of mice. After 4 months from hHSPC injection mice reach full engraftment. Data showing mean and SD from one representative experiment, each dot represents one mouse.

D. Quantification of different hematopoietic cell lineages reconstituted from $TET2^{WT}$ or $TET2^{Mut}$ HSPCs. Data showing mean and SD from one representative experiment, each dot represents one mouse. Unpaired t test used for significance;
* $p<0.05$; ** $p<0.01$.

E. Quantification of TET2-CH by showing variant allele frequency of TET2 mutations in human $CD45^+$ cells from bone marrow of each mouse. Data showing percentage of each TET2 genetic variant in the pool of $TET2^{WT}$ and $TET2^{Mut}$ HSPCs mix injected and in each humanized mouse after 4 months.

F. Quantification of $TET2^{Mut}$-derived clonal hematopoiesis by showing variant allele frequency overtime of TET2 mutations in human $CD45^+$ cells from bone marrow of mice. Human HSPCs from four mice were injected into secondary recipients and no significant differences in the TET2 VAF of the human hematopoietic system was detected after three months of the secondary transplant.

G. Cytometric bead array to quantify the presence of human cytokines in the bone marrow serum of humanized mice (see Figure 3A). Data showing mean from five humanized mice in each group. Each dot represents one mouse whenever cytokines were detected, otherwise non-detected (n.d.) is represented in the plot.

H. Representative flow cytometry gating strategy to analyze $TET2^{WT}$ and $TET2^{Mut}$ HSPCs from the same mouse in untreated (grey panels) or LPS-treated (green panels) humanized mice.

I. Representative flow cytometry gating strategy to analyze different $TET2^{WT}$ and $TET2^{Mut}$ myeloid subsets from the same mouse in untreated (grey panels) or LPS-treated (green panels) humanized mice.

Supplementary Fig S4

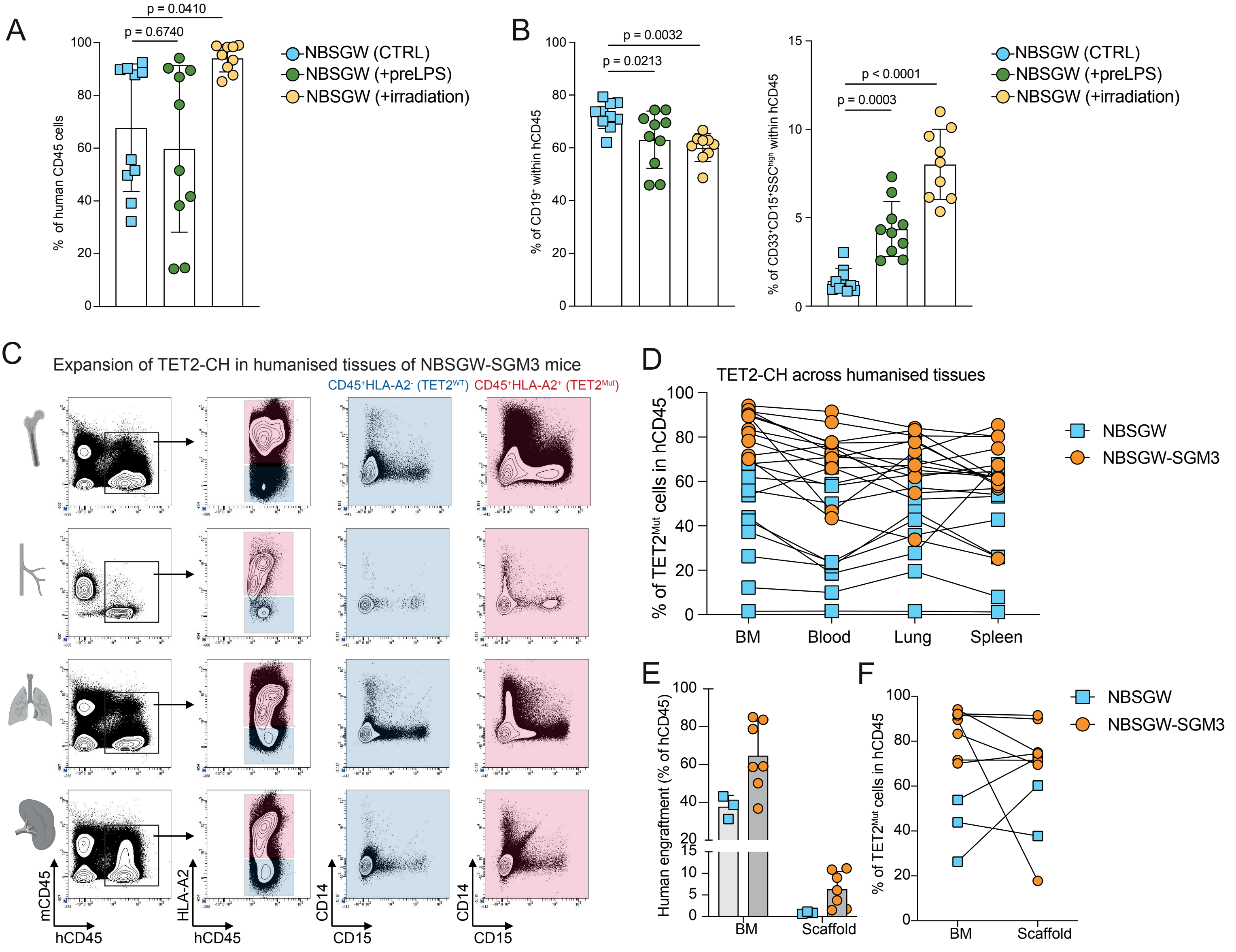

G

In vivo environments triggering emergency myelopoiesis are associated with human TET2-CH increase

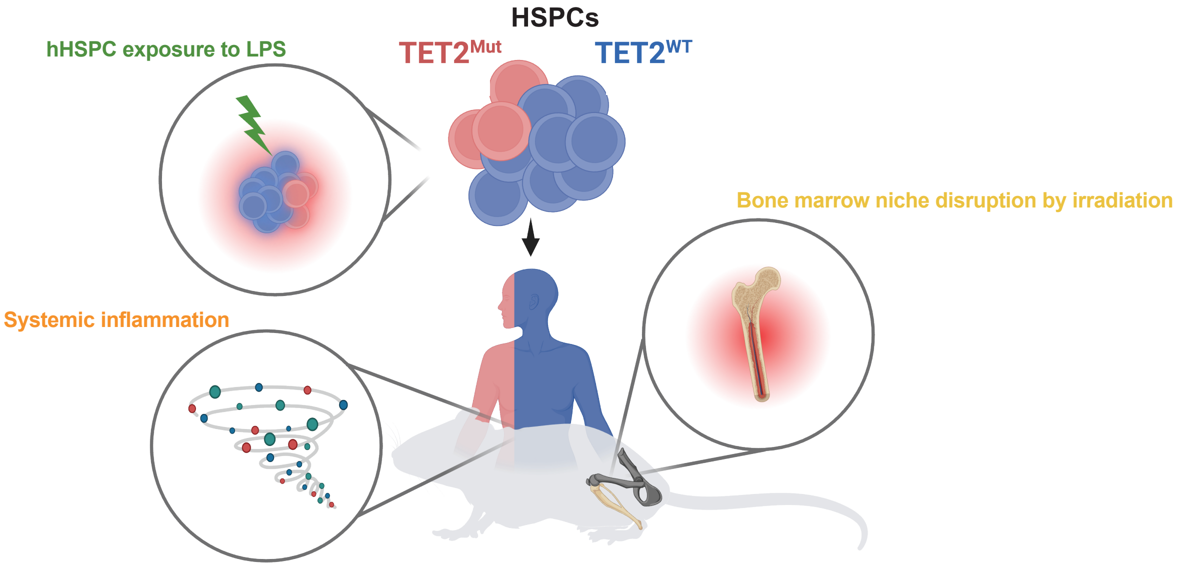

Figure S4. In vivo models triggering emergency myelopoiesis are associated with TET2-CH expansion, related to Figure 3.

A. Quantification of the human engraftment in the bone marrow of mice after 16 weeks from the HSPC injection. Data showing mean and SD from 9-10 humanized mice in each group, each dot represents one mouse. Data from two independent experiments. Two-way ANOVA test used for significance, p-values are shown in the figure.

B. Percentage of B cells (left panel) and neutrophils (right panel) within the human engraftment ($CD45^+$ cells) in the bone marrow. Data showing mean and SD from 9-10 humanized mice in each group, each dot represents one mouse. Data from two independent experiments. Two-way ANOVA test used for significance, p-values are shown in the figure.

C. Representative flow cytometry gating strategy to analyze human engraftment in humanized tissues from NBSGW-SGM3 mice. Mice were injected with a mix (1:1 ratio) of $TET2^{WT}$ and $TET2^{Mut}$ human HSPCs with the HLA-based competition system to allow characterization by flow cytometry of the wild-type and TET2-mutant progeny within the same mouse.

D. Percentage of $TET2^{Mut}$ cells in the human hematopoietic system reconstituted in each tissue to show consistent level of TET2-CH across the tissues from the same mouse. Each line of connected dots represents one mouse (see Figure 3G).

E. Percentage of overall human hematopoietic system reconstituted in the bone marrow and pre-implanted scaffolds of the same mice. Same mix of $TET2^{WT}$ and $TET2^{Mut}$ HSCs in competition was injected in both NBSGW and NBSGW-SGM3.

F. Quantification of $TET2^{Mut}$-derived clonal hematopoiesis in the bone marrow and pre-implanted scaffolds of the same mice (see Figure S3E). Same mix of $TET2^{WT}$ and $TET2^{Mut}$ HSCs in competition was injected in both NBSGW and NBSGW-SGM3. Each line of connected dots represents one mouse to exemplify how TET2-CH expands in both bone marrow and scaffolds of NBSGW-SGM3 compared to NBSGW.

G. Schematic representation of the different environments reproduced in humanized mice that cause an expansion of TET2-CH. The figure was created with Biorender.

Supplementary Figure S5

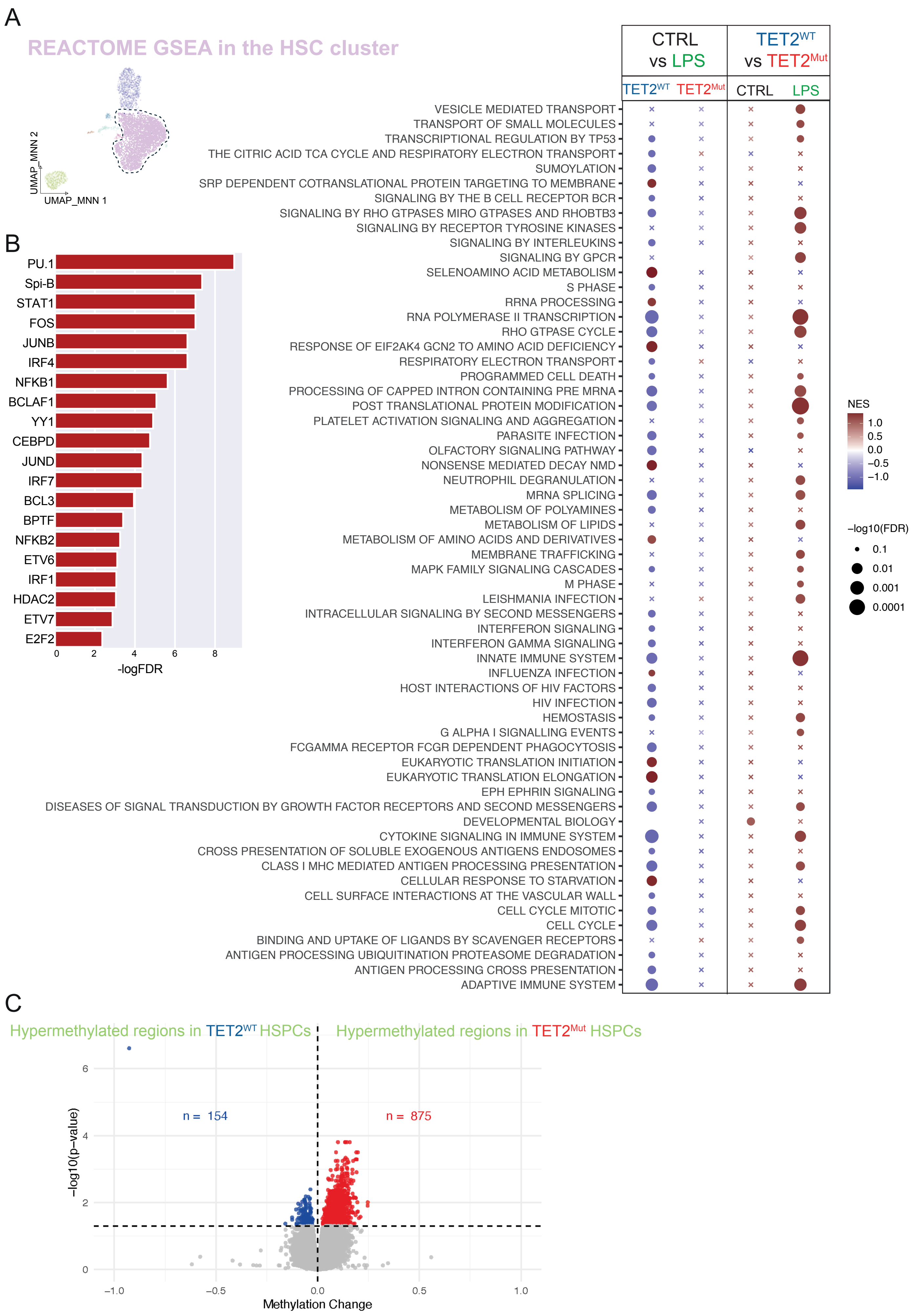

Figure S5. In an in vivo model of systemic inflammation, human TET2^{Mut} HSCs exhibit a dampened transcriptional response, originated from steady-state epigenetic repression of JUN and FOS related to Figure 4.

A. GSEA using Reactome database for the different comparisons displayed in the column header. Only transcriptional pathways that were significant for one of the comparisons are displayed in the figure. Transcriptional pathways with no significant enrichment for a given comparison are represented with a cross.

B. Top 20 differentially activated transcription factors between TET2^{WT} and TET2^{Mut} HSCs cluster in LPS-treated humanized mice. See Supplementary table 4 for the complete list of transcription factors.

C. Volcano plot showing the differentially methylated regions between TET2^{WT} and TET2^{Mut} HSPCs at steady-state. See Supplementary table 5 for the complete list of methylated regions.

Supplementary Figure S6

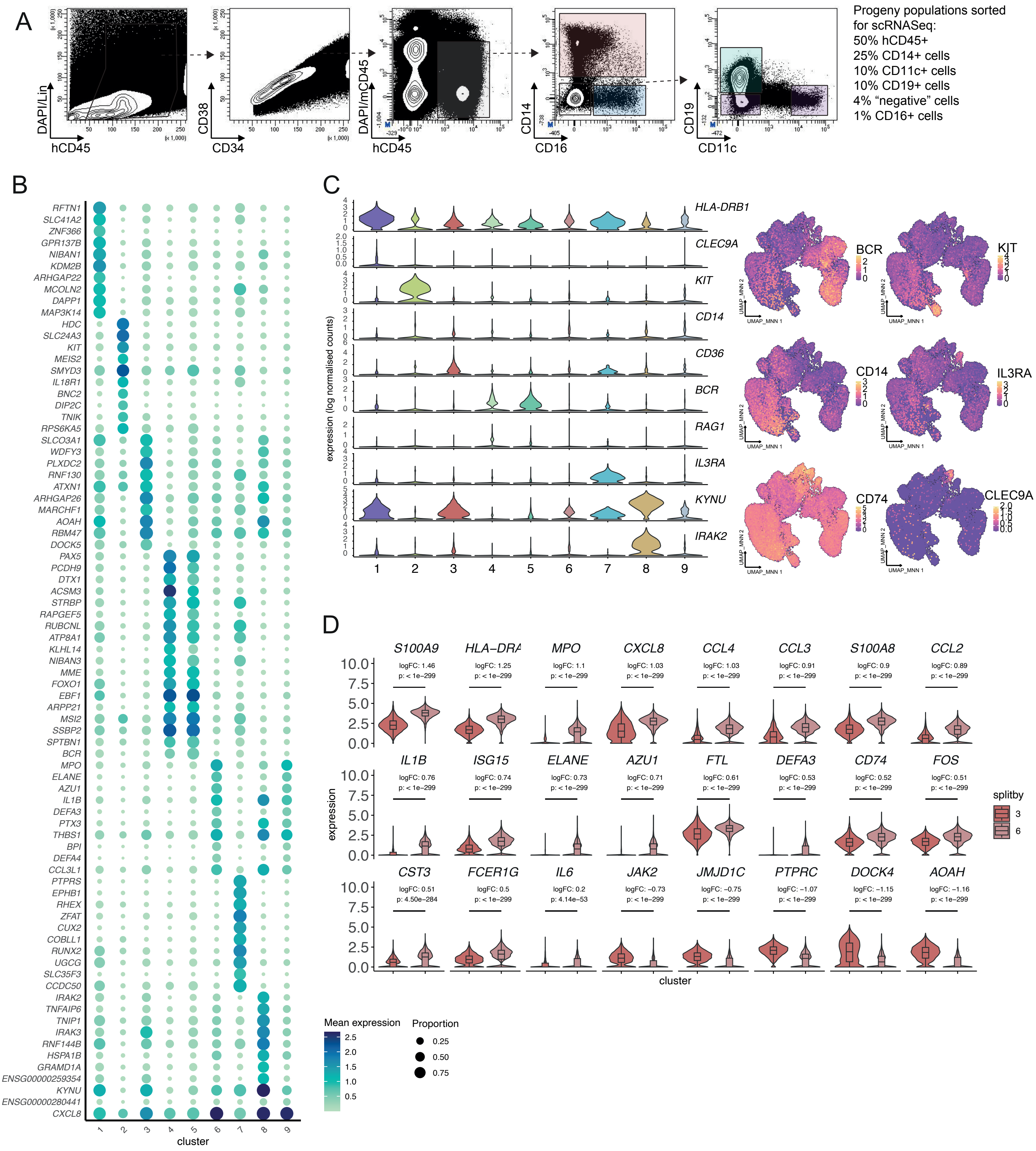

Figure S6. $TET2^{Mut}$ human HSPCs produce a distinct progeny with myeloid cell subsets prompted to produce exacerbated inflammatory response, related to Figure 5.

A. Representative flow cytometry gating strategy to sort immune progeny derived from $TET2^{WT}$ or $TET2^{Mut}$ HSPCs. Pre-fixed percentage of different cell populations were sorted together to ensure enough representation of myeloid subsets and to remove neutrophils as recommended by the manufacturer.

B. Top 10 markers associated with each cell cluster identified in Figure 4F.

C. Expression of canonical genes associated with different immune cell subsets in the clusters identified in Figure 4F.

D. Violin plots of the differentially expressed genes between $CD14^+$ cluster #3 (associated with $TET2^{WT}$ progeny) and $CD14^+$ cluster #6 (associated with $TET2^{Mut}$ progeny). See Figure 4I. Log2 fold change and p-values are displayed in the figure.

Supplementary Figure S7

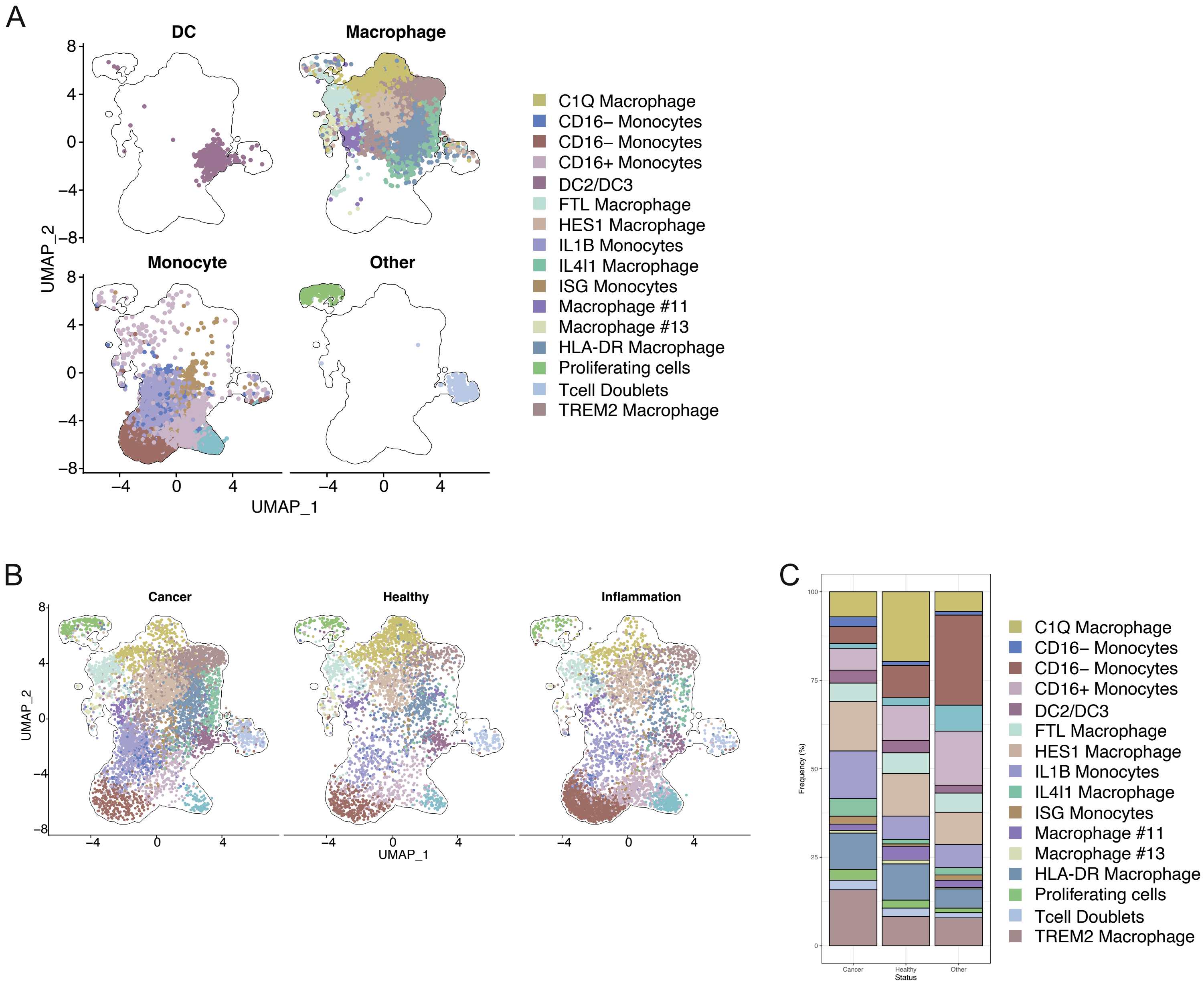

Figure S7. TET2^{Mut} myeloid progeny acquired characteristics of inflamed tissue monocytes and distinct macrophage differentiation, related to Figure 5.

A. UMAP for dimension reduction representation of dendritic cell (DC), macrophage, monocyte and other cell subsets to visualize the space each cell signature occupies in the MoMac-VERSE (see Figure 4K).

B-C. UMAP for dimension reduction representation of the MoMac-VERSE in healthy and different disease context and (C) quantification in stacked bar chart of the different cell signatures for each condition.
